# Phage evolutionary relationships emerge from protein language model-based proteome representation

**DOI:** 10.1093/nargab/lqaf134

**Published:** 2025-10-22

**Authors:** Swapnesh Panigrahi, Mireille Ansaldi, Nicolas Ginet

**Affiliations:** Phage cycle and bacterial metabolism team - Laboratoire de Chimie Bactérienne - UMR7283 CNRS/Aix-Marseille Université, Marseille 13009, France; Phage cycle and bacterial metabolism team - Laboratoire de Chimie Bactérienne - UMR7283 CNRS/Aix-Marseille Université, Marseille 13009, France; Phage cycle and bacterial metabolism team - Laboratoire de Chimie Bactérienne - UMR7283 CNRS/Aix-Marseille Université, Marseille 13009, France

## Abstract

Viral taxonomy is a challenging task due to the propensity of viruses for recombination and the lack of universal gene markers. As a result, recent ICTV updates increasingly rely on multiple tools for taxonomic ranking, with a growing emphasis on proteome-based clustering approaches. At the same time, the rapid expansion of viral datasets presents new challenges in organizing, analysing, and discovering phage relationships at scale. To address these challenges, we introduce hierarchical viruses, a framework for comparative genomics of bacteriophages that leverages protein Language Model (pLM) embeddings to generate proteome-wide vector representations of phages. Clustering the vector representations of 24 362 phages from the curated INPHARED dataset reveals a multi-scale hierarchical organization of phages. This hierarchy aligns with current ICTV taxonomic rankings at the genus and subfamily levels, with an adjusted mutual information score greater than 0.9 for both, in the *Herelleviridae* family, demonstrating that pLM-based proteome representations can effectively capture evolutionary relationships without relying on multiple sequence alignments. The framework builds the basics towards vectorial phage datasets that encode evolutionary information, thus allowing discovery of phage relationships at scale.

## Introduction

Self-supervised Large Language Models based on transformer architecture have been successful in encoding contextual information content of a word (or more generally a token) in a sentence. Various methods have been developed using the learnt vector representations of the tokens (or embeddings) for classification of sentences and further for clustering of texts [1]. Similarly, protein Language Models (pLMs) treat amino-acid sequences as tokens and, due to availability of large datasets of proteins, have been trained in a self-supervised manner to learn contextual information and evolutionary fitness of amino-acids in a sequence. This information is encoded in the embeddings, which are vectorial representations of this information in an abstract continuous space. Such embeddings have been used for downstream tasks like protein annotation [2, 3]. The embeddings and attention maps from different layers of the model have been shown to also encode structural information of a protein [4–6]. There are currently few such pre-trained pLMs available to the public, for example Evolutionary Scale Modelling 2 (ESM-2) or ProtT5-XL-U50 [7, 8]. These models are trained on the large protein UniRef50 dataset [9], thus capturing the evolutionary fitness of proteins in the embeddings. ESM shows that protein representations extracted from the pre-trained pLM cluster into groups with similar structure and align with previously annotated protein families [7]. Thus, proteins that are closer in the protein representation space are likely to be functionally similar.

We posit that protein representations generated from pLMs can be extended to entire proteomes to compare and classify biological entities. By proteome, we mean the set of distinct proteins that is encoded by any single biological entity’s genome (e.g. a eukaryotic cell, an archaeon, a bacterium or a virus). One can view a proteome as the summation of functions acquired through evolution and one way to apprehend evolutionary relationships between biological entities can be done by comparing their proteomes.

Here we focused on a particular class of biological entities, the viruses of bacteria (bacteriophages or phages) that are defined by rather small proteomes compared to bacterial, archaeal, and eukaryotic ones. Phages are diverse in terms of nucleic acid supporting the genetic information (e.g. DNA, RNA, single-stranded, or double-stranded), their gene content as well as the range of bacterial hosts they infect. Phages have been extensively studied for more than a century and there exists an abundant scientific literature covering all aspects of phage studies. The INPHARED database (September 2024 release) contains 29 759 reportedly complete, annotated prokaryotic virus genomes [10]. This number is increased by a factor 30 with metagenomic data in the PhageScope database [11]. Comparing and classifying such a high and exponentially increasing number of genomes is a key issue in the field. Phage taxonomy is a challenging task owing to the propensity of phages for recombination between viruses or with their hosts. Phage genome organization is described as mosaic, with sets of genes regrouped in modules related to certain functions such as capsid or tail assembly, genome replication, or cell lysis [12–14]. Such modules can be swapped between phages through recombination, rendering phage evolutionary history particularly challenging. Typical examples are lambdoid phages exemplified by the famous *Escherichia coli* phage Lambda. Furthermore, in contrast with other biological entities, there aren’t any universally shared genetic traits among viruses such as the 16S rRNA gene in bacteria. Viral taxonomy has to rely on multiple tools with different results as illustrated by Barylski *et al.* study of the phage *Herelleviridae* family and manual curation of taxonomy experts [15].

The current viral classification including bacteriophages is kept separated from other biological entities and maintained by the ICTV [16]. The long history of phage classification reflects progresses in phage biology and investigation techniques over a century. This classification is currently undergoing a profound overhaul enabled by whole genome and proteome pipeline analyses with on-going efforts towards a unified taxonomy [17]. State-of-the art viral comparative genomics relies now on whole genome and proteome approaches such as vConTACT v2.0, VipTree or GRAVITy [18–20]. The ICTV classification has undergone a profound reshaping in 2022 enabled by the above-mentioned approaches [21]. At the time of this manuscript's preparation (December 2024), prokaryotic viruses included in the INPHARED database (September 2024 release) were spread among five realms. The majority of these (79.4%) are double-stranded DNA viruses (*Duplodnaviria* realm) equipped with a tail (*Caudoviricetes* class). Thanks to metagenomically identified viruses, we can confirm that many taxa in reference databases such as INPHARED are underpopulated due to historical and methodological biases. Hence recent metatranscriptomic studies of soil and ocean communities revealed unsuspected diversity among single-stranded RNA phages (*Riboviria* realm) [22, 23]. Starting with four species classified in two genera, the ICTV ratified in 2021 the creation of 428 genera and 882 species. The minimal ITCV requirement for every virus is a species and a genus affiliation. While 95% nucleotide sequence identity over the genome length is widely accepted as a demarcation criterion for species across the different viral realms, criterion for genus demarcation is highly variable. For instance, phage genera belonging to the *Inoviridae* family (*Monodnaviria* realm) are inferred from CoatB and Zot protein phylogenies (ICTV proposal 2016.080jB). For tailed, double-stranded DNA viruses infecting bacteria and archaea (*Caudoviricetes* class), 70% nucleotide identity over the genome length is generally accepted as the genus demarcation criterion. Nevertheless, whole genome DNA comparisons usually fail to capture evolutionary relationships to define taxonomic ranks above genus. Subfamily and family demarcation criteria are still non-homogenous as they depend on available data, bioinformatic analyses available at the time of their definition and their various combinations by different authors. As an example, the *Autographivirinae* subfamily—promoted at the family level in 2021 as *Autographiviridae*—has been proposed in 2009 on the following criteria: *encode their own RNA polymerase and share a common general genome organization, with genes encoded solely on the Watson strand* (ICTV proposal 2008.020–023B.v3). This definition is biased towards a single, specific biological activity (the RNA polymerase). The *Ackermannviridae* family and its internal structure relies on DNA sequence identity, shared prokaryotic virus orthologous groups (pVOG) and single protein phylogenies (terminase large subunit and tail-sheath proteins) studies (ICTV proposal 2017.001B). To the best of our knowledge, the most comprehensive study for phage family definition is exemplified in the above-mentioned work of Barylski et *al*. [15]_._ Using a double-stranded DNA (dsDNA) phage similarity network generated with vConTACT v2.0, the authors proposed a new taxon for a group of 93 dsDNA tailed phages, the *Herelleviridae* family. By combining genome-based and proteome-based analyses, gene synteny analysis and marker proteins phylogenies, the authors could refine the internal structure of this new taxon into five defined subfamilies and 15 genera. This work is another illustration of the on-going effort to clarify taxon demarcations in phage taxonomy using genome-wide approaches.

The revolution enabled by massive parallel DNA sequencing during the past 10 years and the ensuing development of metagenomics resulted in an exponentially increasing number of available viral sequences. As an illustration, the online bacteriophage database PhageScope registers 873 718 bacteriophage genomes and 43 088 582 annotated proteins (November 2024 release) [11]. These numbers cannot but increase exponentially as new environments are being intensively sampled and their viromes sequenced. Hence, there is a crucial need for new comparative genomics approaches to grasp the prokaryotic virosphere in a more global, systematic and scalable way.

We present here an approach we named hierarchical viruses (HieVi), which uses pretrained pLMs for whole proteome clustering. HieVi is not a supervised classification model. Instead, it leverages the representational power of pLMs to capture functional and evolutionary signals across phage proteomes in an unsupervised manner. Since phage comparative genomics often relies nowadays on shared protein functions, we hypothesized that a simple average over all protein representations from a phage proteome encodes the information about distribution of protein functions within that proteome. HieVi encodes each phage proteome by a single vector, a mean phage representation (MPR). We generated MPRs for the 24 362 complete and annotated prokaryotic viral genomes present in the HieVi and clustered them using a density-based clustering algorithm. This allowed us to construct a HieVi hierarchical tree computed from the pairwise Euclidean distance matrix between MPRs. We observed that the HieVi hierarchical tree topology revealed multi-scale taxonomic relationships, assisting in the interpretation and refinement of existing classifications. From the largest perspective to the finer grain, we observed that viruses first cluster in the tree according to the realm and kingdom they belong to, then that phages belonging to the same ICTV family are, in many cases, clustered in the same branch and finally that phages generally also cluster within this branch according their ICTV genus and subfamily annotations. We showed that HieVi topology is in good accordance with established phage phylogenies at multiple scales. We documented these observations with a few known phage families and provided an example of how HieVi can be used to refine current taxonomy and correct inappropriate assignments. We finally propose that HieVi can help define new phage taxa and discover unsuspected evolutionary relationships. In a broader perspective, this study is the first example of pLM implementation to compare biological entities. HieVi can be extended to more complex organisms such as bacteria, archaea or eukaryotes, computational resources, biological diversity, and expert validation being the only limits for this.

## Materials and methods

### Viral proteome dataset

We used the September 2024 release of the INPHARED prokaryotic viral database maintained and regularly updated by Millard's lab [10]. This dataset comprises 29 759 annotated genomes, mostly from the RefSeq and GenBank databases. We filtered this database for unique accessions, resulting in the HieVi dataset with 24 362 genomes and 2 166 614 proteins. The authors regularly curate their database from incomplete genomes. To ensure the best possible consistency across the dataset, they also performed gene calling on their entire database with the same gene caller. This dataset includes mostly phages but also viruses infecting archaea and unclassified hosts. It also covers a wide range of proteome sizes (Table 1).

**Table 1.** HieVi dataset. (**A**) Number of viruses sorted by their prokaryotic host super-kingdom. (**B**) Proteome size distribution (number of CDS per genome). Total number of genomes *n* = 24 362 included in the INPHARED database (September 2024 release)

(A) Prokaryotic host			(B) Proteome size		
Bacteria	Archaea	Unclassified	<10	50–100	>200
20 995 (86.2%)	295 (1.2%)	3075 (12.6%)	14.2%	47.7%	13.0%

This dataset covers all taxonomic ranks present in the current ICTV prokaryotic viral taxonomy (Table 2).

**Table 2.** Taxonomic description of the HieVi dataset. Taxonomic ranks are defined by the ICTV (as defined in December 2024) and displayed in hierarchical order (the realm being the highest taxonomic rank). The four top annotated taxa were displayed for each taxonomic rank. ICTV accepted proposals defining current viral taxa are available through the ICTV website (https://ictv.global/taxonomy). Orange: *Duplodnaviria* viruses. Green: *Monodnaviria* viruses. Blue: *Varidnaviria* viruses. Violet: *Riboviria* viruses

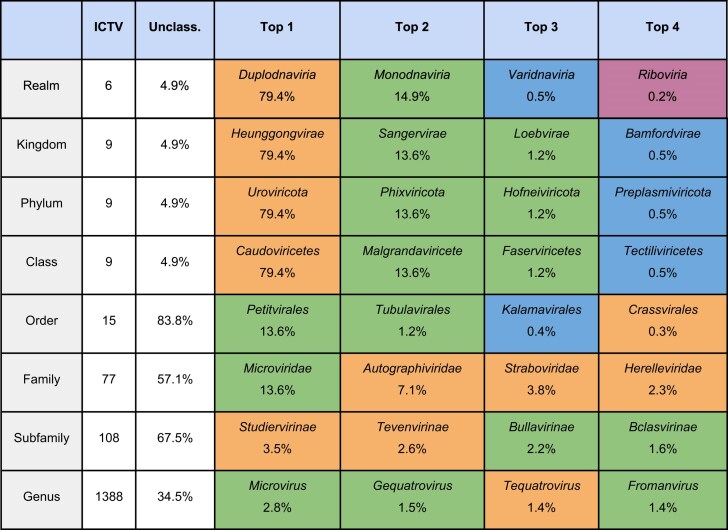

Table 2 illustrates the current challenges in viral taxonomy with about ∼57% unclassified viruses at the family level and ∼34% at the genus level. This table also points at several sampling biases inherent to current phage genome databases. For instance, dsDNA tailed-phages (*Caudoviricetes* class) represent ∼79% of INPHARED genomes whereas few single-stranded RNA (ssRNA) phages (*Riboviria* realm) are recorded (0.2%). This is mostly due to historical reasons (e.g. focus on human pathogenic bacteria), practical reasons (*e.g*. usage of cultivable lab strains for phage isolation) or methodological reasons (e.g. sequencing techniques favouring dsDNA viruses). These biases are being progressively reduced, thanks to metagenomic and metaviromic approaches on samples from extraordinarily diverse environments (*e.g*. oceans, glaciers, clouds, sediments, or various microbiota).

### Phage similarity network generated by vConTACT v2.0

We generated with the HieVi dataset a phage similarity network using state-of-the-art vConTACT v2.0 pipeline based on shared PCs [18]. Diamond was used for protein sequence all-versus-all alignments [24]. Protein clusters (PCs) and viral clusters (VCs) were generated by MCL and ClusterOne, respectively. All other parameters were set to default values. The 2 166 614 viral protein sequences included in our dataset were first grouped into 125 242 PCs within which proteins are expected to perform similar functions. Among the 24 362 viruses in the dataset, 20 784 were clustered into 3209 VCs where viruses are grouped according to PCs they share. The remaining 3578 genomes either overlap with two VCs (*n* = 2 044 genomes) or were defined as singleton (*n* = 311 genomes) and outliers (*n* = 1 223 genomes) by the pipeline. vConTACT v2.0 results for each accession in the HieVi dataset are summarized in the Supplementary Excel file HieVi_annotations.xlsx.

### Pre-trained pLM ESM-2

Evolutionary Scale Modelling 2 (ESM-2) is a pLM that leverages transformer architectures to learn amino-acid embeddings capturing biochemical and functional information within a protein [7]. It has been shown that ESM-2 learns protein representations that encode the evolutionary and structural properties of proteins without explicit structural data. The protein representations align with a protein family and function, making them useful for downstream functional classification tasks such as predicting sub-cellular localization and catalytic activity.

In this study, we used the ESM-2 pre-trained model (esm2_t36_3B_UR50D) which has been trained on the dense and highly diverse protein dataset Uniref50 (UR50, version 2021/04) available at the UniProt database (https://www.uniprot.org/). This pre-trained model currently contains 3 billion parameters (ESM-2 3B), has 36 layers with an embedding dimension of 2560.

Our starting viral dataset consisted in 2 166 614 protein sequences and necessitated sufficient GPU memory (∼24–32 GB for ESM-2 3B) to load the model and perform inference efficiently. For this purpose, we utilized a NVIDIA A40 GPU (with GPU memory 48 GB), which is necessary for efficient computation using the 3 billion parameter ESM-2 model. Additionally, to facilitate efficient processing of this large proteomic dataset, it was stored in the Zarr format codified by the Open Geospatial Consortium (https://www.ogc.org/).

### HieVi Phage representation

To generate a vector representation of an entire phage proteome, we treat a phage as a bag of constituent proteins denoted by the set $\Phi = \{ {{{u}_1},{{u}_2},\ \ldots,\ {{u}_m}} \}$ with ${{u}_i}$ being the normalized protein embeddings from ESM-2 such that $| {| {{{u}_i}} |} | = 1$. The protein representations are computed by mean pooling over the amino-acid embeddings from the ESM-2 and then normalizing into unit vectors. Then, we define the MPR as the average of all protein representations within each proteome, symbolically, $\phi = \frac{1}{m}\mathop \sum \limits_{i = 1}^m {{u}_i}.$

Protein embeddings are normalized before averaging to ensure that each protein function contributes equally to the angular (functional) space, preventing highly conserved proteins (which might otherwise have larger norms) from disproportionately dominating the mean. The MPRs are thus vector representations of phage's proteomes within the same 2560-dimensional space as the protein embedding representation from ESM-2 3B. We generated MPRs for all unique accession numbers in the HieVi dataset, resulting in 24 362 vectors.

### HieVi distances and metrics

Euclidean distance between MPRs is used as the metric for comparing phages and for clustering. Symbolically, the Euclidean distance between phages ${{\Phi }_u}$ and ${{\Phi }_v}$ is given by $| {| {\ {{\phi }_u} - {{\phi }_v}} |} |$. A mathematical formulation of the construction of the MPRs is provided as a supporting document in Supplementary Information II. The biological interpretations and reasoning for the choice of Euclidean distance is discussed in the following sections.

Silhouette score is used to quantify the clustering validity of phage representations of the ICTV genera and subfamilies annotations within annotated ICTV families [25]. For external validation according to the ICTV genus/subfamily annotations or vConTACT v2.0 VC clusters, we used adjusted mutual information (AMI) [26, 27].

### Clustering phage representations

For clustering the 24 362 MPRs, we used Hierarchical Density-Based Spatial Clustering of Applications with Noise (HDBSCAN), a clustering algorithm that builds on the DBSCAN (Density-Based Spatial Clustering of Applications with Noise) framework [28, 29]. This allows for MPRs clustering based on density with a possibility of extracting flat clusters for a distance threshold. This distance threshold for extracting flat DBSCAN clusters is denoted as eps. Visualization of the high-dimensional phage representation in a two-dimension Phage Atlas was performed using Uniform Manifold Approximation and Projection (UMAP) [30]. UMAP preserves the local manifold structure, which is useful for illustrating the genus-level clusters, thus it is solely used for visualization in the 2D phage atlas. Other dimension reduction methods like tSNE [31] could also be used for the visualization. We do not use dimensionality reduction for core clustering and distance calculations which are rather performed on the original high dimensional space to avoid distorting the biological relations captured by the original learnt embeddings.

### Phage networks visualization

We used Cytoscape 3.10.3 [32] to visualize vConTACT v2.0 phage similarity network (with yFiles organic layout) and HieVi hierarchical tree (with the free release of yFiles Hierarchical Layout algorithm from yWorks GmbH). The annotation file provided with the INPHARED dataset comprises for each virus accession number numerous information such as the current viral taxonomy, isolation host, genome length or number of tRNA genes. We enriched this table, whenever possible, with partial host taxonomy, host envelope descriptor (Gram positive, Gram negative, or other descriptors) or virion morphology. We also extracted from protein annotations several keywords such as integrase, excisionase, RNA polymerase, etc. We added in this table for each accession number vConTACT v2.0 results as well as our own categories discussed in several figures in this manuscript. This HieVi annotation table is used in Cytoscape to search and investigate HieVi hierarchical tree topology on various criteria and provided as Supplementary Excel File HieVi_annotation.xlsx.

## Results

### HieVi phage atlas: phage clusters align with annotated genus

**Figure 1.**
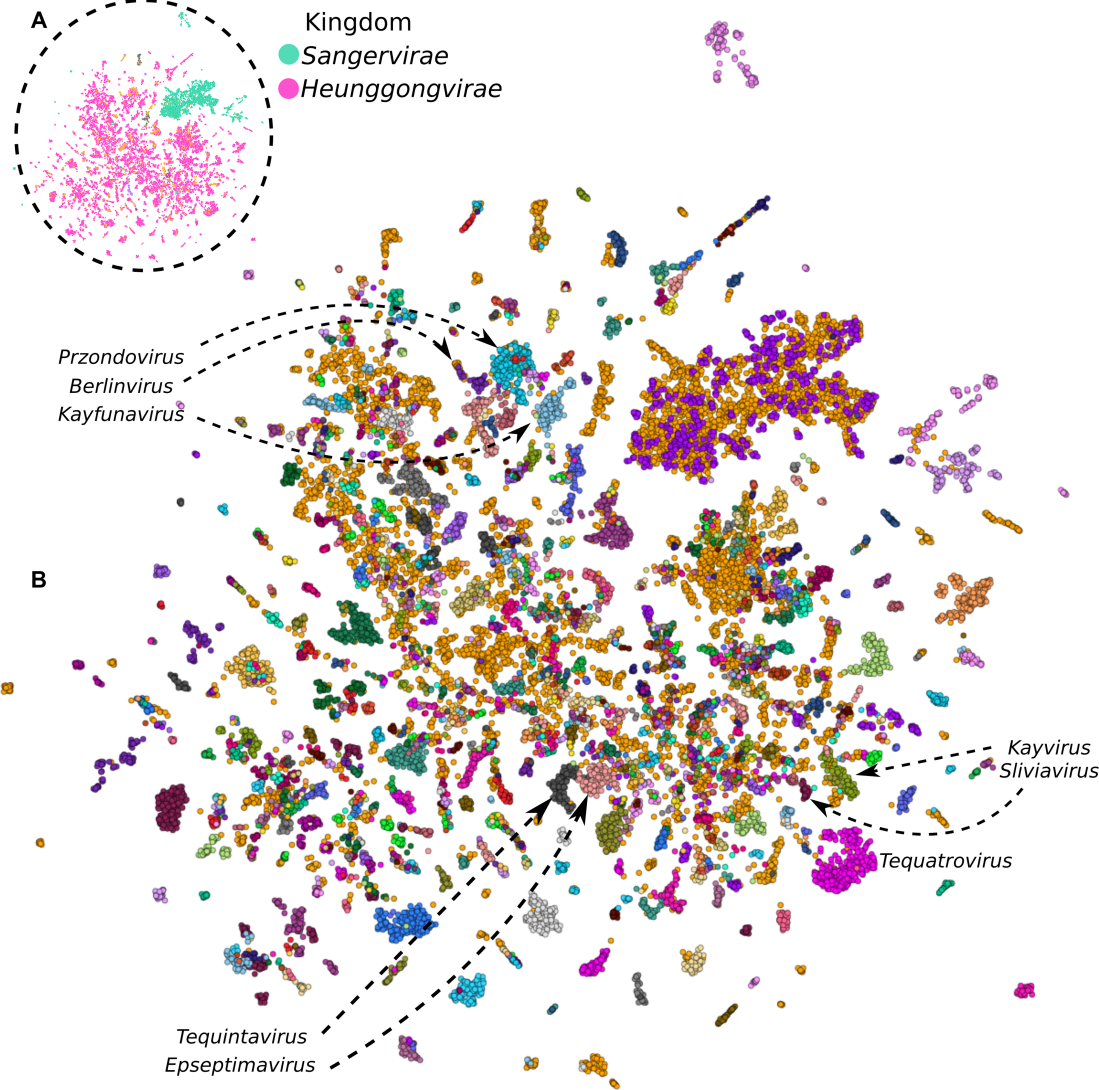
HieVi phage atlas of the hievi dataset. Visualization of the phage representations (*n* = 24 362 viruses) in two dimensions. (**A**) Viruses coloured by ICTV kingdoms. *Heunggongvirae* and *Sangervirae* ICTV kingdoms were highlighted in pink and green, respectively. Unclassified viruses are coloured in orange. (**B**) Viruses coloured by ICTV genera. Pointed clusters: *Przondovirus*, *Berlinvirus* and *Kayfunavirus* (*Autographiviridae* family, *Studiervirinae* subfamily); *Tequintavirus* and *Epseptimavirus* (*Demerecviridae* family, *Markadamsvirinae* subfamily); *Silviavirus* and *Kayvirus* (*Herelleviridae* family, *Twortvirinae* subfamily); *Tequatrovirus* (*Straboviridae* family, *Tevenvirinae* subfamily). Unclassified viruses are coloured in orange. An interactive HieVi Phage Atlas (September 2024 release) is available (see Data availability section). UAMP parameters: n_neighbors = 16 and min_dist = 0.6.

The 24 362 MPRs vectors representing all viruses in the HieVi dataset in the 2 560 dimensions space were projected on a two-dimensional HieVi Phage Atlas to visualize the viral diversity using UMAP (Fig. 1).

Each point in this atlas is a phage genome from the HieVi dataset coloured by an ICTV taxonomic rank. In Fig. 1A, phages are coloured by ICTV kingdoms. We observe that viruses are well clustered according to the realm they belong to, with *Heunggongvirae* and *Sangervirae* forming the two major clusters in the Phage Atlas. *Heunggongvirae* (dsDNA phages coding for a HK97-fold capsid protein) is the only kingdom from the *Duplodnaviria* realm (dsDNA phages) and encompasses the totality of the *Caudoviricetes* class (tailed phages), the most abundant group of phages in our dataset. *Sangervirae* (single-stranded DNA or ssDNA prokaryotic viruses coding for a single jelly roll fold capsid protein) is one of the four kingdoms of the *Monodnaviria* realm (ssDNA phages). In Fig. 1B, viruses are coloured by ICTV genera. On the finer grain, well separated clusters can be observed that are consistent with the ICTV genus level classification in most cases. For example, *Tequatrovirus* forms a well separated cluster. *Epseptimavirus* and *Tequintavirus*, the two genera defining the *Markadamsvirinae* subfamily, form two separate clusters but remain close in the approximated manifold of MPRs. The clustering efficiency of the MPRs with respect to ICTV genus is quantified by an average Silhouette score of 0.30 [25]. The average is over all the phages in the dataset (even those with a single representation of the phage in the genus and family). The histogram of Silhouette score per sample is given in Supplementary Information I in Supplementary Fig. S1. We also plot in this Figure the mean Silhouette score of phages by family to show that indeed the MPRs cluster well with ICTV genus. Silhouette score ranges from −1 to 1 and a score greater than zero indicates a fairly good clustering of MPRs at the genus level. It is worth keeping in mind that the unclassified viruses are ignored and that we rely on the ICTV genus annotation.

HieVi Phage Atlas suggests that MPRs embedded multiscale information for classification of phages well-above the genus level. The ICTV genus is well defined by a single criterion, i.e. >70% nucleotide identity across the entire genome. With such a threshold, phage proteomes are thus expected to share a significant number of orthologous proteins within a given genus. It is thus unsurprising that the MPRs cluster together by genus as these phage representations contain information about accumulated protein functions within a proteome. It is less obvious for subfamilies as they are not defined by strict criteria such as nucleotide identity but emerge from a combination of approaches such as gene/protein sharing networks or single/multiple gene/protein phylogenies.

### Construction of MPR and biological interpretation

**Figure 2.**
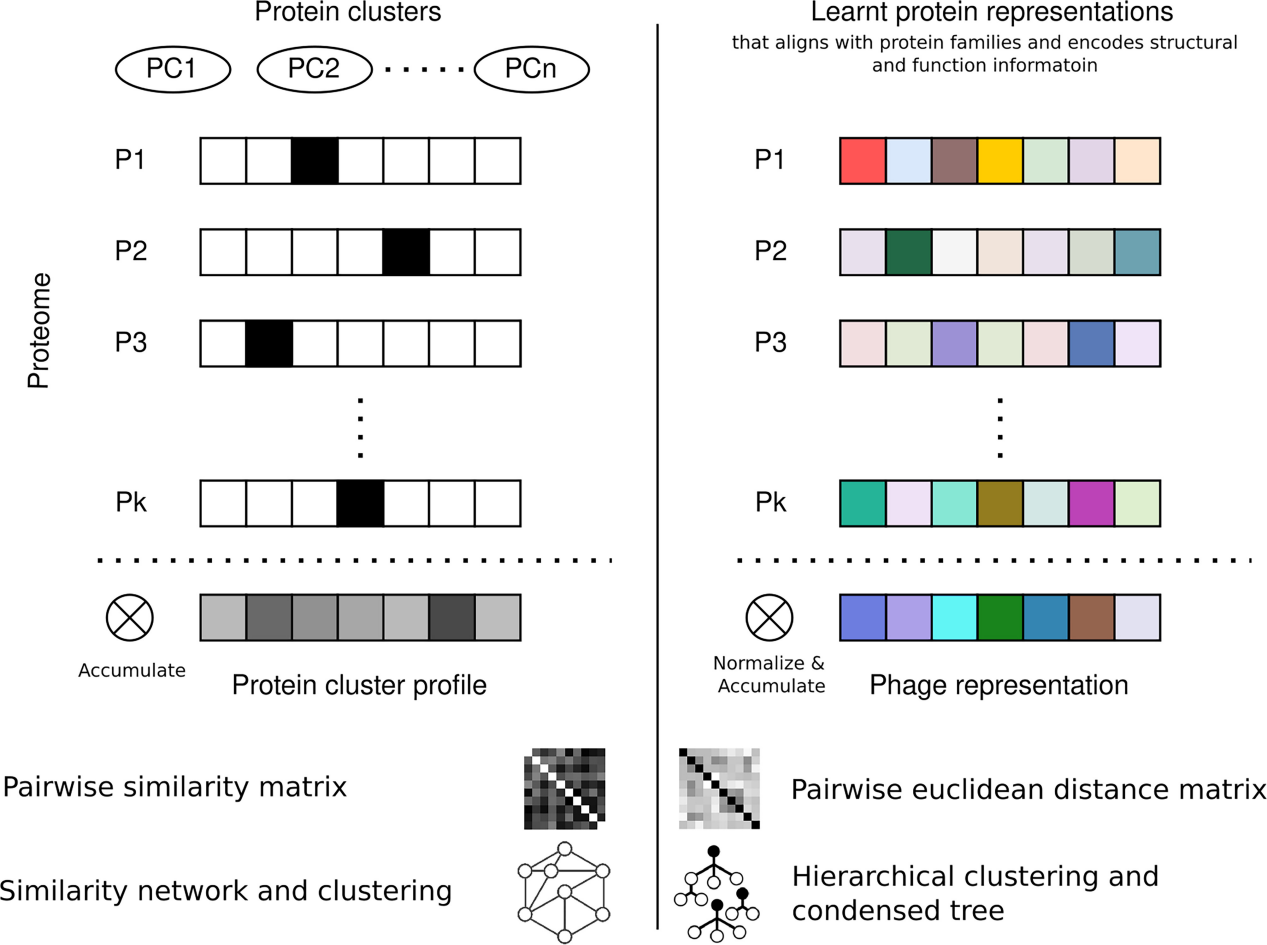
Conceptual parallels between vConTACT v2.0 and HieVi. On the one hand, in vConTACT v2.0, each protein may be seen as a one-hot vector in the PC space with dimension *n* equal to the number of PCs (*n* = 25 513 in the initial vConTACT v2.0 paper. Then, the PC profile for each genome is generated by summing over all the vectors. A similarity matrix is then computed using the hypergeometric formula (taking into account the genome lengths) and then a dense similarity network is generated and clustered using ClusterOne. On the other hand, in HieVi the protein representation generated using a pre-trained pLM has been shown to align with protein families and encodes protein function, structure and evolutionary fitness. Averaging over the protein representations in a proteome then gives a unique phage representation. A pairwise Euclidean distance matrix between all viruses is computed, then hierarchically clustered using HDBSCAN and a tree-like hierarchical network is finally generated.

To understand why the simple averaging of all protein representations within a phage proteome leads to well-defined phage clusters in the HieVi Phage Atlas, we formalize the construction of the MPRs in the Supplementary Information II and drew, in Fig. 2, conceptual parallels with the methodology employed by vConTACT v2.0, a bioinformatic pipeline that cluster viruses according to their shared protein content [18].

As mentioned previously, vConTACT v2.0 pipeline first generates PCs with MCL. Then, for each proteome, PC profiles were defined by accumulating the shared PCs. This is equivalent to representing proteins as one-hot vectors in the PC space and then accumulating the proteins within a proteome to obtain a PC profile which is a vector of size of the number of PCs (*n*= 125 242 in this study with the HieVi dataset). A pairwise similarity matrix was then computed using these vectors and a similarity network was generated. Network clustering by ClusterOne was finally used to identify well separated clusters. Notably, the number of PCs increases in vConTACT v2.0 together with the number of genomes (*n* = 2 304 genomes and *n* = 25 513 PCs in the initial vConTACT v2.0 paper, *n* = 24 362 genomes and *n* = 125 242 PCs in the present study).

Computational time and store scaling of vConTACT v2.0 is reported to be linear with number of genomes [18]. However, adding a single (or few) genome requires re-computation of the PCs using vConTACT v2.0. In contrast, HieVi requires one-time computation of the entire database with computational time comparable to vConTACT v2.0, whereafter, analysis of new genomes by nearest neighbour search requires less than a second (depending on genome size and length of the constituent proteins). Moreover, continuously adding complete genomes to the vector database can only increase the accuracy of analysis without computationally costly one-to-all comparisons. In our framework, identification of a finite number of PCs is not necessary since the protein embeddings are shown to clusters along protein families [7]. Based on this observation, we formalize the construction of the MPRs and provide a detailed mathematical description in the Supplementary Information II. We assume that a normalized protein embedding ${{u}_i}$ may belong to a protein family cluster $k( i )$ having a corresponding direction given by the unit vector ${{\mu }_{k( i )}}$. Each cluster have a compactness parameter given by ${{\alpha }_{k( i )}}$. Then, we derive the expected squared norm of each representation and dot-product between the MPRs. The biological interpretations inferred from the formalism is presented below:

#### Norm of MPRs reflect functional diversity

We derive in Equations [1–5] of the Supplementary Information II, the expected squared-norm of the MPRs under our framework, given by


\begin{eqnarray*}
&& E[\left|| \phi |\right|^2]\ = \frac{1}{m}\ + \frac{1}{{{{m}^2}}}\mathop \sum \limits_{i \neq j}^{} {{\alpha }_{k\left( i \right)}}{{\alpha }_{k\left( j \right)}}{{\mu }_{k\left( i \right)}}^T{{\mu }_{k\left( j \right)}}
\end{eqnarray*}

The expected norm of the MPR serves as a proxy for functional diversity within a phage genome. When protein embeddings come from the same or closely related families, they align in angular space, resulting in relatively larger norms. In contrast, when embeddings span unrelated families, their directions are more dispersed, leading to a smaller norm. In contrast to random unit vectors (i.e. no learned structure), where the norm tends to decrease with increase in the number of vectors being averaged, embeddings from pre-trained pLMs remain compact in angular space, maintaining a non-negligible norm even with diverse protein inputs.

#### Dot-product between MPRs reflect shared protein content

Similarly, we derive in Equations [6–9] of the Supplementary Information II the dot-product between two phages ${{\Phi }_u} = \{ {{{u}_1},{{u}_2},\ \ldots,\ {{u}_{{{m}_u}}}} \}$ and ${{\Phi }_v} = \{ {{{v}_1},{{v}_2},\ \ldots,\ {{v}_{{{m}_v}}}} \}$ as,


\begin{eqnarray*}
&& E\left[ {{{\phi }_u}^T\ {{\phi }_v}} \right]\ = \frac{1}{{{{m}_u}{{m}_v}}}\left( {\mathop \sum \limits_{k = 1}^K {{n}^u}_k{{n}^v}_k{{\alpha }_k}^2\ \ + \ \mathop \sum \limits_{k = 1}^K \mathop \sum \limits_{l = 1}^K {{n}^u}_k{{n}^v}_l{{\alpha }_k}{{\alpha }_l}{{\mu }_k}^T{{\mu }_l}} \right),
\end{eqnarray*}

where, ${{n}^u}_k$ and ${{n}^v}_l$ are the number of proteins in phage ${{\Phi }_u}$ belonging to a family $k$ and the number of genes in phage ${{\Phi }_v}$ belonging to a family $l$, respectively. The first term (with $k$ equal to $l$) is the number of shared proteins (same cluster in the embedding space) weighted by the compactness of the protein family they belong to. The second term ($k$ not equal to $l$) describes the shared protein families that are close in the protein embedding space. This second term is smaller when the protein families are further apart in the embedding space.

#### Euclidean distance measures both intra-genomic function diversity and inter-genomic similarity

The expected squared Euclidean distance between two phages can be written as: $E[|| {{{\phi }_u} - \ {{\phi }_v}} |{{|}^2}] {\ = E} [\parallel {{\phi }_u}{{\parallel }^2}] { + \ E} [\parallel {{\phi }_v}{{\parallel }^2}] { - 2E} [{{\phi }_u}^T\ {{\phi }_v}],$ thus allowing us to interpret the information embedded in the Euclidean distance. As shown before (and derived in the Supplementary Information II), the norm of the MPRs encodes the functional diversity and the dot-product encodes the shared protein information. Therefore, the Euclidean distance is a measure of both functional diversity and protein families shared by the two phages under consideration. Using this formulation, we may define other metrics that reflect other biological criteria for phage comparison.

In our framework, identification of a finite number of PCs isn’t necessary since the embeddings already encode protein family information in the entire space of the proteins in the ESM-2 training dataset. As a consequence, HieVi requires a one-time computation of MPRs from a reference dataset. Then, comparing new genomes only requires computation of new MPR and a nearest neighbour search on the pre-generated MPRs dataset. Thus, this framework is well-suited for leveraging advancements in vector databases and fast nearest-neighbour algorithms [33] for scalable genomic comparison. In the next sections, we employ hierarchical clustering on the MPRs generated on the INPHARED database and extract a tree of hierarchical relations between phages in the database. We show that the MPRs encode biological and evolution information in the embedding space and multi-scale taxonomic relations emerge.

### HieVi hierarchical clustering and tree

**Figure 3.**
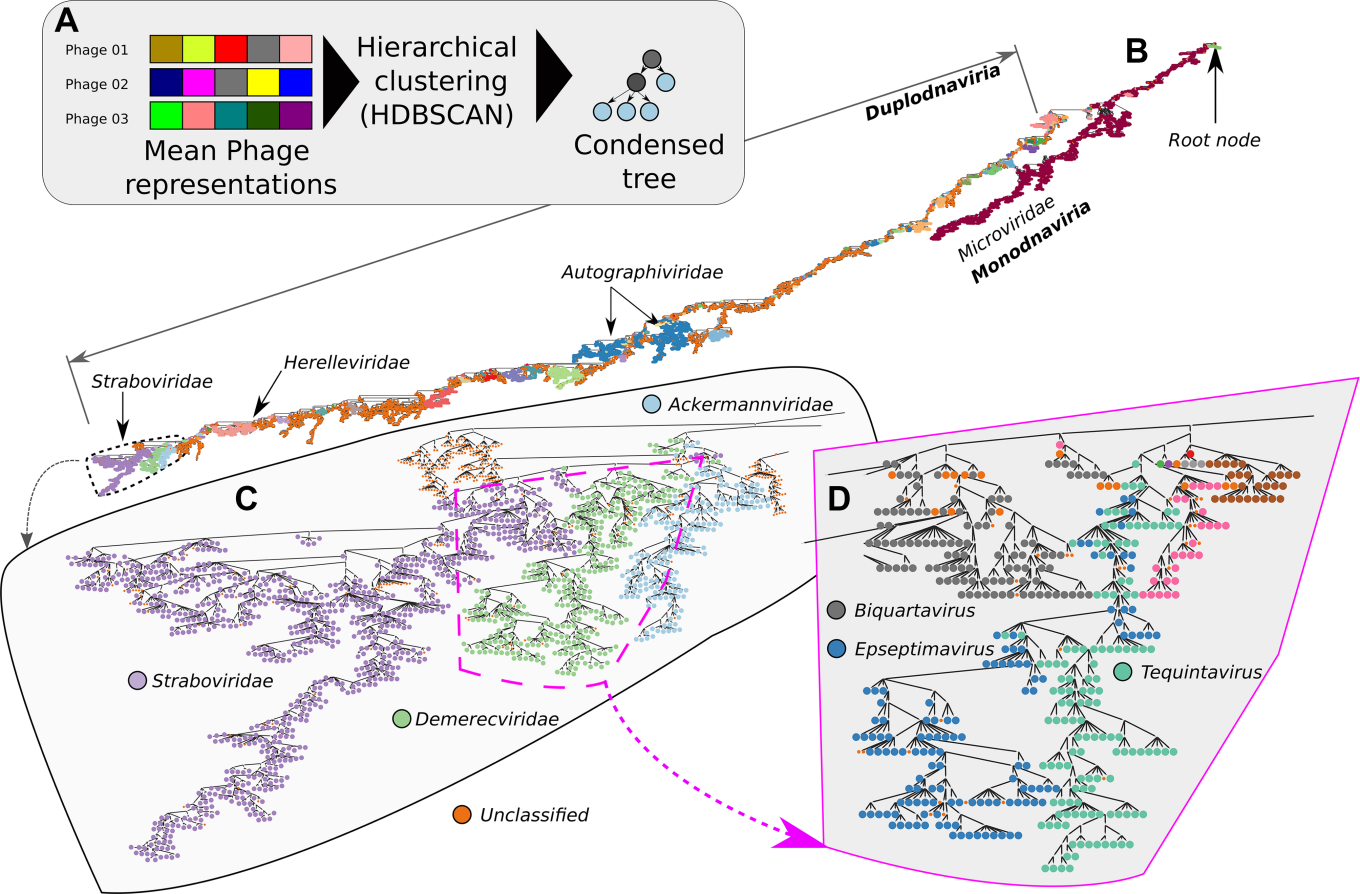
HieVi hierarchical network. (**A**) Workflow to generate the HieVi hierarchical tree from the MPRs. Network hierarchy and topology show multiscale phage groupings, coherent with the ICTV realm (*Duplodnaviria* and *Monodnaviria* in (**B**), family (*Demerecviridae*, *Straboviridae* and *Ackermannviridae* in (**C**) and genus (*Biquartavirus*, *Epseptimavirus* and *Tequintavirus* in (**D**). Unclassified viruses (family or genus) are displayed in orange in Panels B to D. Relevant annotations are summarized in the Supplementary Excel file HieVi_annotations.xlsx. The HieVi hierarchical tree network (Supplementary Zip file HieVi_hierarchical_tree.zip) can be viewed in Cytoscape.

We used HDBSCAN with the Euclidean distance metric between vectors to obtain a granular cluster so that there are at least two leaves per cluster (parameter minPts = 2), essentially constructing a dendrogram. HDBSCAN is designed to handle data with varying cluster densities and is particularly useful for identifying clusters of varying shapes and sizes without knowing the number of clusters *a priori* [29]. We then extracted the condensed tree that HDBSCAN uses to cluster the 24 362 viruses as our hierarchical tree of phages (Fig. 3).

Fig. 3B shows an overview of the large network containing all virus proteomes in the dataset as a hierarchical tree. At the top of the hierarchy one can find the *Monodnaviria* (ssDNA phages) realm that transitions into phages from other realms (not indicated) and finally into *Duplodnaviria* (dsDNA phages). The large network is coloured by ICTV families with orange representing unclassified viruses. For aid, we indicated *Autographiviridae*, *Herelleviridae* and *Straboviridae* families that we chose to study in further detail. On a finer scale, we observe in Fig. 3C that phages are grouped in separated branches corresponding to the ICTV families. Finally at the finest grain, Fig. 3D shows that phages cluster with respect to the ICTV genus. At least qualitatively, we see multiscale phage grouping emerging from the HieVi hierarchy and topology, which constitute a clarifying step beyond the vConTACT v2.0 Phage network for the entire dataset (Supplementary Fig. S2).

To further investigate the meaning of this topology, we first focused on the *Straboviridae* and *Herelleviridae* families. These two families were defined quite recently using whole genome approaches (see ICTV proposal 2021.082B for *Straboviridae* and Barylski *etal*. for *Herelleviridae* [15]. *Straboviridae* phages infect mostly *Gammaproteobacteria* belonging to *Enterobacterales*, *Aeromonadales*, *Moraxellales* and *Vibrionales* orders whereas *Herelleviridae* infect bacteria mostly belonging to *Bacillales* and *Lactobacillales* orders. Both families form several well-defined VCs in close proximity in the vConTACT v2.0 Phage Network (Supplementary Fig. S2).

For each of these two families, we identified in the HieVi hierarchical tree the top node of phages annotated for the corresponding ICTV family and extracted all its successors. We thus obtained one branch regrouping 94.8% of the *Straboviridae* phages and one branch regrouping 94.2% of the *Herelleviridae* phages. These two branches are described in Table 3.

**Table 3.** Straboviridae and Herelleviridae branch annotations. *Straboviridae* branch comprises 37 ICTV genera (64 phages are unclassified at the genus level). *Herelleviridae* branch comprises 36 ICTV genera (97 phages are unclassified at the genus level)

Branch	ICTV / Unclassified	Subfamily	Main host orders
*Straboviridae* (946)	878 / 68	*Guernseyvirinae* (1), *Tevenvirinae* (615), *Twarogvirinae* (34), Unclassified (296)	*Enterobacterales* (834), *Moraxellales* (42), *Aeromonadales* (40), *Vibrionales* (26)
*Herelleviridae* (517)	528 / 43	*Andregratiavirinae* (2), *Bastillevirinae* (149), *Brockvirinae* (51), *Jasinskavirinae* (36), *Joanripponvirinae* (9), *Sejongvirinae* (7), *Spounavirinae* (27), *Twortvirinae* (264), Unclassified (26)	*Bacillales* (517), *Lactobacillales* (53),

Fig. 4 displays the *Straboviridae* and *Herelleviridae* HieVi branches. We extracted as well the corresponding vConTACT v2.0 networks. Phages in both HieVi branches and vConTACT v2.0 networks were coloured according to the ICTV genus.

**Figure 4.**
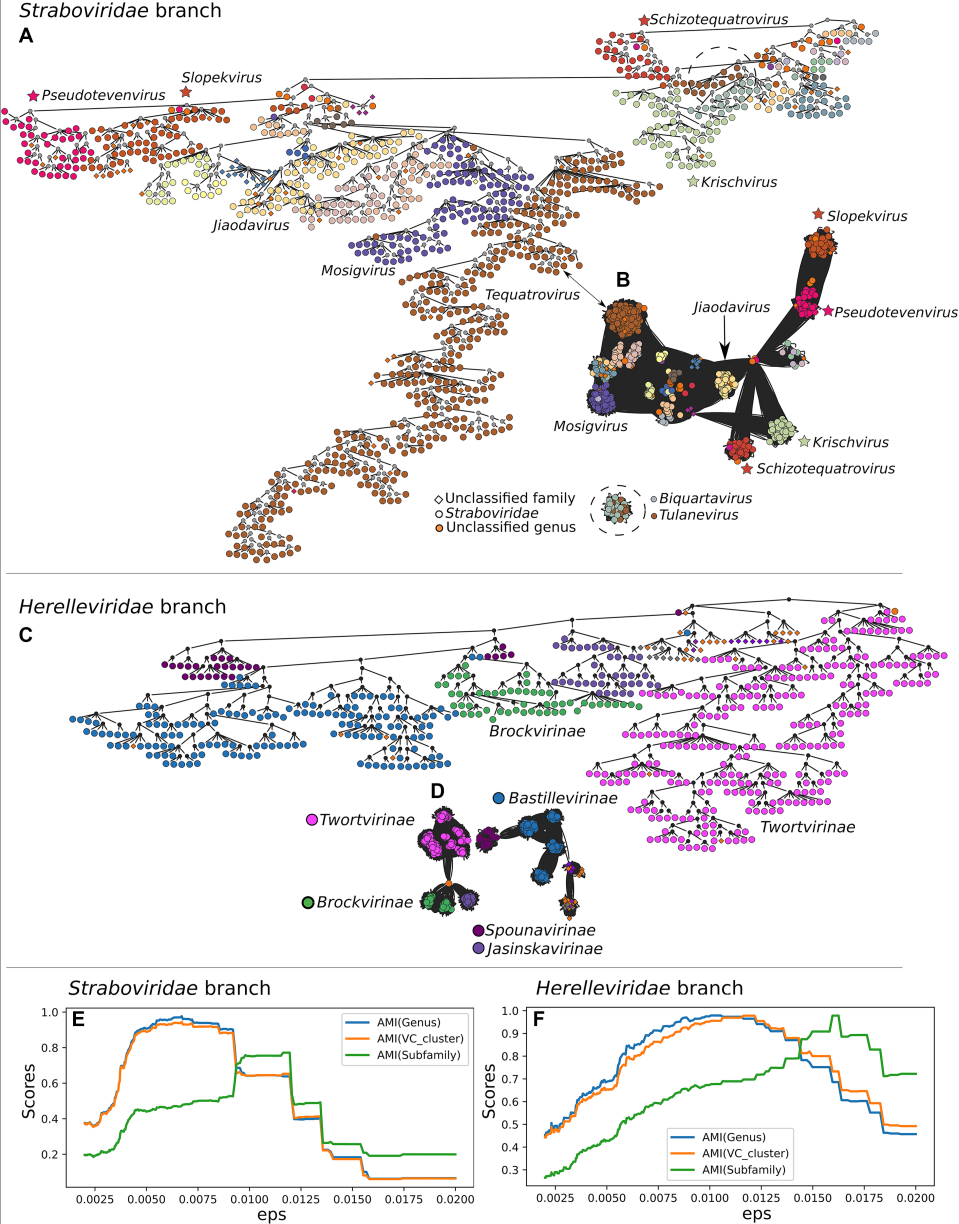
*Straboviridae* (left) and *Herelleviridae* (right) families. (**A**) HieVi branch for *Straboviridae* phages, coloured by ICTV genera. (**B**) vConTACT v2.0 network for *Straboviridae* phages, coloured by ICTV genera. (**C**) HieVi branch for *Herelleviridae* phages, coloured by ICTV subfamilies. (**D**) vConTACT v2.0 network for *Herelleviridae* phages, coloured by ICTV subfamilies. Some genus or subfamily correspondence between HieVi branches and vConTACT v2.0 networks are indicated by arrows. (**E**, **F**) AMI scores plotted against eps distances for ICTV genus (blue curves) and for ICTV subfamily (green curves) for HieVi branches, for VC annotation (orange curve) for vConTACT v2.0 networks. (**E**) AMI scores for *Straboviridae* phages. (**F**) AMI scores for *Herelleviridae* phages. Relevant annotations are summarized in the Supplementary Excel file HieVi_annotations.xlsx.

Fig. 4 shows that phages are well clustered in the HieVi hierarchical tree according to the ICTV genera (*Straboviridae*, Fig. 4A) and the ICTV subfamilies (*Herelleviridae*, Fig. 4C). This observation is quantitatively supported by the clustering efficiency metrics displayed in Fig. 4E (*Straboviridae*) and Fig. 4F (*Herelleviridae*). The AMI scores with respect to the ICTV genera (blue curves) and subfamilies (green curves) are > 0.8, indicative of an efficient clustering with respect to the annotations for these two families (AMI score varies from 0 to 1). The AMI scores were computed by comparing ICTV annotations with cluster labels obtained for each flat cluster at each eps. We obtained the same performance for other phages families (Supplementary Fig. S3A). The AMI score profiles with respect to vConTACT v2.0 VCs (orange curves) are highly similar to the ones obtained with HieVi with the ICTV genus annotation, which is expected as it has been shown that VCs generally cluster well phages at the genus level. At variance with HieVi, vConTACT v2.0 VCs do not cluster phages at the subfamily level. For instance, *Bastillevirinae* subfamily phages are grouped in the *Herelleviridae* HieVi branch (highlighted in blue in Fig. 4C) while they are separated into four VCs (VC_196, VC_197, VC_199, and VC_953, highlighted in blue in Fig. 4D) in the vConTACT v2.0 network.

AMI score profiles displayed Fig. 4E and F show that one can modulate the eps distance threshold to cluster phages at the genus or the subfamily level within a given family. Nevertheless, these AMI profiles also show that the eps value is not the same for a given taxonomic rank across all phage families. It is thus not possible to define a single eps threshold to predict genera (or subfamilies) across the entire HieVi hierarchical tree (Supplementary Fig. S3B).

As an intermediary conclusion, we evidenced that HieVi enables a representation of the phage world as we know it as a hierarchical tree that can capture multiscale relationships. On the broader view, viruses are rather well clustered according to the realm they belong to. We observed that viruses belonging to the same ICTV family remain generally close in the tree topology. We also qualitatively and quantitatively evidenced that HieVi is efficient in clustering viruses at two successive taxonomic ranks, *i.e*. genus and subfamily.

#### Case study: Autographiviridae

According to ICTV, *Autographiviridae* is a phage taxon regrouping podovirus phages encoding their own single large subunit RNA polymerase with all genes transcribed on the Watson strand. Inclusion within this ICTV family is mostly defined by these two criteria (see ICTV proposal 2008.020–023B). *Autographiviridae* is a particularly illustrative example of the challenges in phage classification as well as how the HieVi tool could be useful in this context to biologists in the field for *Autographiviridae* phages taxonomy.

**Figure 5.**
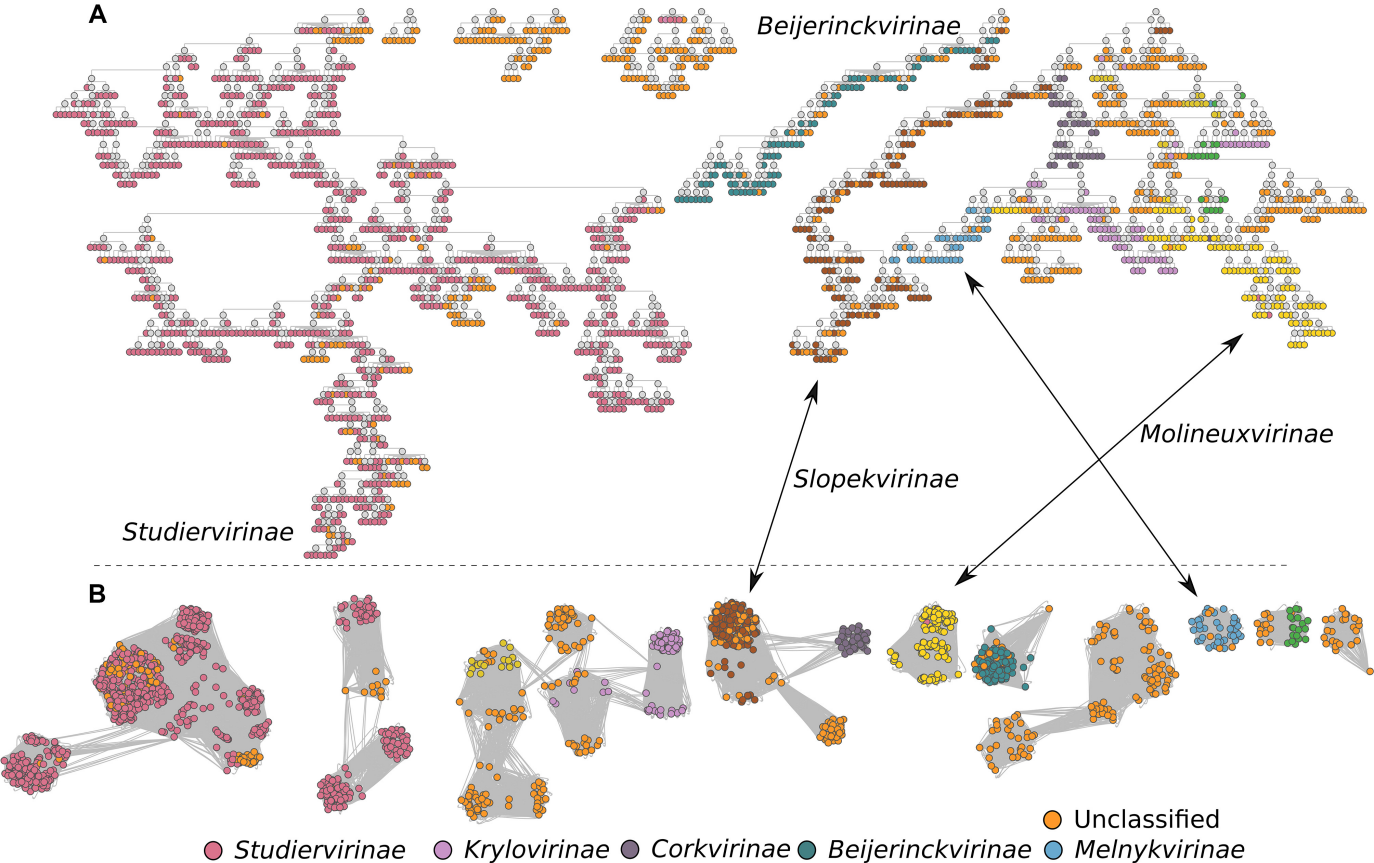
*Autographiviridae* family. (**A**) HieVi branches for *Autographiviridae*. (**B**) vConTACT v2.0 network for *Autographiviridae*. Phages are coloured by ICTV subfamilies. Relevant annotations are summarized in the Supplementary Excel file HieVi_annotations.xlsx.

Our dataset comprises 1 743 *Autographiviridae* viruses assigned to eight subfamilies and 130 genera in the current ICTV classification, evidencing that this family is very diverse. In vConTACT v2.0 Phage network, *Autographiviridae* phages form several, unconnected clusters (Supplementary Fig. S2, highlighted in blue). At variance with the phage families we previously investigated, *Autographiviridae* phages are not clustered in the same branch in the HieVi hierarchical tree. Around 96.7% of these phages are spread among nine branches (01 to 09, Table 4), with 01 and 02 branches accounting for 83.6% of the *Autographiviridae* dataset. The corresponding HieVi branches and vConTACT v2.0 networks are displayed in Fig. 5 with viruses coloured by ICTV subfamilies.

**Table 4.** *Autographiviridae* branches annotations. These nine branches comprise in total 121 ICTV genera (382 phages remain unclassified at the genus level). ^#^Number of phages within each group/taxon is displayed within brackets. *Phages belong to another ICTV family. Total number of phages *n* = 1 874

Branch^#^	*Autographiviridae* / Unclassified / Other*	Subfamily^#^	Main host orders^#^	Predicted integrase Yes / No	Genome size (kb)
01 (952)	858 / 94 / 0	*Studiervirinae* (843), Unclassified (110)	*Enterobacterales* (827), *Pseudomonadales* (68), *Vibrionales* (25)	1 / 951	39.7 ± 1.2
02 (671)	600 / 70 / 1	Bclasvirinae** (1), *Colwellvirinae* (19), *Corkvirinae* (36), *Krylovirinae* (75), *Melnykvirinae* (36), *Molineuxvirinae* (108), *Okabevirinae* (17), *Slopekvirinae* (127), *Studiervirinae* (2), Unclassified (250)	*Enterobacterales* (392), *Pseudomonadales* (84), *Xanthomonadales* (50), *Vibrionales* (45), *Burkholderiales* (34), *Aeromonadales* (27), *Hyphomicrobiales* (23), *Caulobacterales* (11)	22 / 649	43.6 ± 1.9
03 (122)	101 / 21 / 0	*Beijerinckvirinae* (91), *Slopekvirinae* (7), Unclassified (24)	*Acinetobacter* (108)	0 / 122	41.5 ± 0.9
04 (50)	50 / 0 / 0	Unclassified (50)	*Prochlorococcus* (29), *Synecococcus* (18)	15 / 35	42.8 ± 4.0
05 (36)	36 / 0 / 0	Unclassified (36)	*Pelagibacter* (30)	31 / 5	40.6 ± 2.0
06 (14)	14 / 0 / 0	Unclassified (14)	*Enterobacterales* (14)	0 / 14	42.0 ± 0.7
07 (14)	14 / 0 / 0	Unclassified (14)	*Rhizobium* (4), *Ralstonia* (8)	14 / 0	41.7 ± 1.5
08 (9)	9 / 0 / 0	Unclassified (9)	*Roseobacter* (9)	9 / 0	40.0 ± 0.9
09 (6)	4 / 1 / 1	*Studiervirinae* (4), Tunavirinae* (1), Unclassified (1)	*Enterobacterales* (4)	0 / 6	48.7 ± 11.8

In the INPHARED dataset (September 2024 release), an annotation error was identified: proteins associated with *Mycobacterium* phage Calamitous (*Bclasvirinae*, *Autographiviridae* branch 02, accession MZ747518) actually belong to *Salmonella* phage vB_SenAt-pSL2 (a *Zindervirus*, accession MZ573924). This was confirmed by BLASTing the MZ747518_00 043 protein, which showed 100% identity with the RNA polymerase of the *Salmonella* phage. *Autographiviridae* branch 09 includes four *Kayfunavirus* genomes (accessions OP272490 to OP272493) whose proteomes differ from other *Kayfunavirus* (usually in branch 01) due to poor syntactic annotation, shown by unusually high numbers of predicted ORFs (83 versus 49 on average) (Supplementary Fig. S5). This likely stems from low-quality DNA sequences resulting in artificial stop/start codons. This example illustrates the importance of correct syntactic annotation—hence protein prediction—since MPR vectors derive from protein embeddings. Additionally, branch 09 includes *Shigella* phage vB_SsoS_008 (accession MK335533), a *Tunavirus* from the *Drexlerviridae* family. This association of this phage with the ill-annotated *Autographiviridae* viruses within branch 09 is difficult to explain other than invoking another erroneous syntactic annotation that spirited this phage away from its *Tunavirus* relatives in the HieVi hierarchical tree.

As was the case for *Straboviridae* and *Herelleviridae*, we observed that *Autographiviridae* phages cluster in the HieVi hierarchical tree according to the ICTV genus (data not shown) but also with the ICTV subfamily (Fig. 5A). Such is not always the case in vConTACT v2.0 phage network. As an example, 843 out of the 856 *Studiervirinae* phages in the dataset are grouped into a single HieVi branch (Fig. 5A) while they are spread among several poorly connected or unconnected VCs in vConTACT v2.0 network (Fig. 5B). HieVi topology suggests new relationships between *Autographiviridae* subfamilies and HieVi branches could be used by experts in the field as a blueprint to investigate new taxa regrouping previously acknowledged ones. HieVi is thus able to capture connections between subfamilies that were previously unlinked.

**Figure 6.**
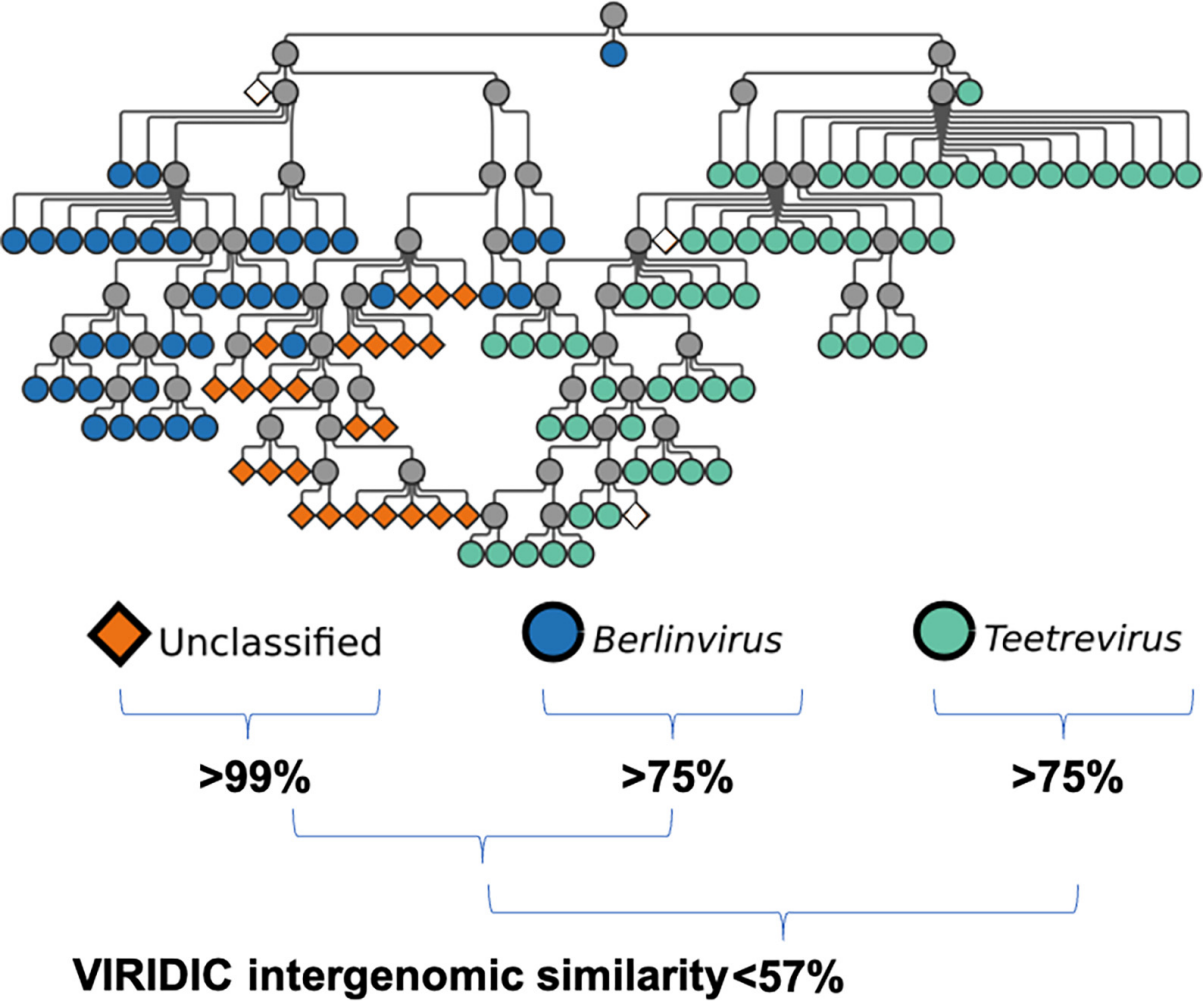
Taxonomic assignment of unclassified phages within the *Autographiviridae* branch 01 (*Studiervirinae*). (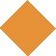) Unclassified family, subfamily and genus. (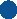) *Berlinvirus*. (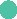) *Tetreevirus*. Intergenomic similarity between groups was computed with VIRIDIC [27]. Relevant annotations are summarized in the Supplementary Excel file HieVi_annotations.xlsx.

On a finer grain, HieVi can help classify unclassified phages in an effective fashion as illustrated in Fig. 6 within *Autographiviridae* branch 01 that mostly includes *Studiervirinae* phages.

In Fig. 6, one can identify a branch comprising 24 phages represented in orange, unclassified at the family, subfamily and genus levels, plugged into the *Berlinvirus* genus branch and adjacent to the *Treetrevirus* branch. Since we previously evidenced that HieVi topology follows genus and subfamily annotations, we posit these unclassified phages are, at the least, *Autographiviridae* belonging to the *Studiervirinae* subfamily, and eventually *Berlinvirus*. We computed with VIRIDIC [34] the intergenomic distances matrix for these 24 unclassified phages and the surrounding *Berlinvirus* and *Tetreevirus* and clustered them with nucleotide identity threshold >95% and >70% for species and genus levels, respectively. VIRIDIC clustering clearly states that these 24 unclassified phages represent a single, *Berlinvirus* species.

As mentioned previously, *Autographiviridae* is a diverse family with eight subfamilies and 130 genera. Combining HieVi topology, functional annotation or other differentiating criteria can also draw attention to new relationships. A first type of connection relates to *Autographiviridae* genome size. The second type derives from the combination of HieVi topology and protein functional annotation. For the latter we examined the presence or absence of a predicted integrase within the proteomes. Table 4 summarizes genome length and predicted integrase for all nine HieVi *Autographiviridae* branches.

*Autographiviridae* genome size distribution is bimodal, with one peak around 40.5 kb and the other around 43.5 kb (Supplementary Fig. S4). In Table 4, we can see that *Autographiviridae* phages are well sorted across the two main branches (01 and 02) according to their genome size. Branch 01 hosts smaller genomes (39.7 ± 1.2 kb) than branch 02 (43.6 ± 1.9 kb). are mostly clustered in two branches (01 and 03), while bigger genomes (>42 kb) are mostly clustered in branch 02. Genome size could thus be used as an additional criterion to improve *Autographiviridae* taxonomy.

Regarding the presence of a predicted integrase protein in *Autographiviridae* proteomes, we can readily observe in Table 4 that three branches (05, 07 and 09) almost exclusively encompass phages harbouring an integrase, an indication of a putative temperate lifestyle. Branch 04 presents a mixed situation with about half of its members encoding an integrase, either due to simple functional misannotation or maybe, more interestingly, due to gain/loss of function.

On a broader perspective, our results suggest that HieVi tree topology can assist experts in the field in refining, improving or even questioning the current structure of the *Autographiviridae* family. Since HieVi branches regrouping subfamilies are consistent with established phylogenies, these branches could serve to define new families encompassing phylogenetically related phages coding for their own large single subunit RNA polymerase. Genome size or the presence of an integrase could also be used as criteria to define membership in these new families. *Autographiviridae* could thus be promoted at the rank order (*Autographivirales*) and include these newly defined families. The inclusion criteria within this new order would be the same as those defined for the current *Autographiviridae* family. Interestingly, during the course of this manuscript preparation, ICTV recently ratified (February 2025) a proposal creating the *Autographivirales* order, comprising four new families (ICTV proposal 2024.045B). In light of this recent development, we provide an updated Table 4 in Supplementary Information I (Supplementary Table S2), which shows for each branch that we had previously defined the corresponding new ICTV annotations.

#### Case study: Discovery of a cohesive group of lambdoid phages

**Figure 7.**
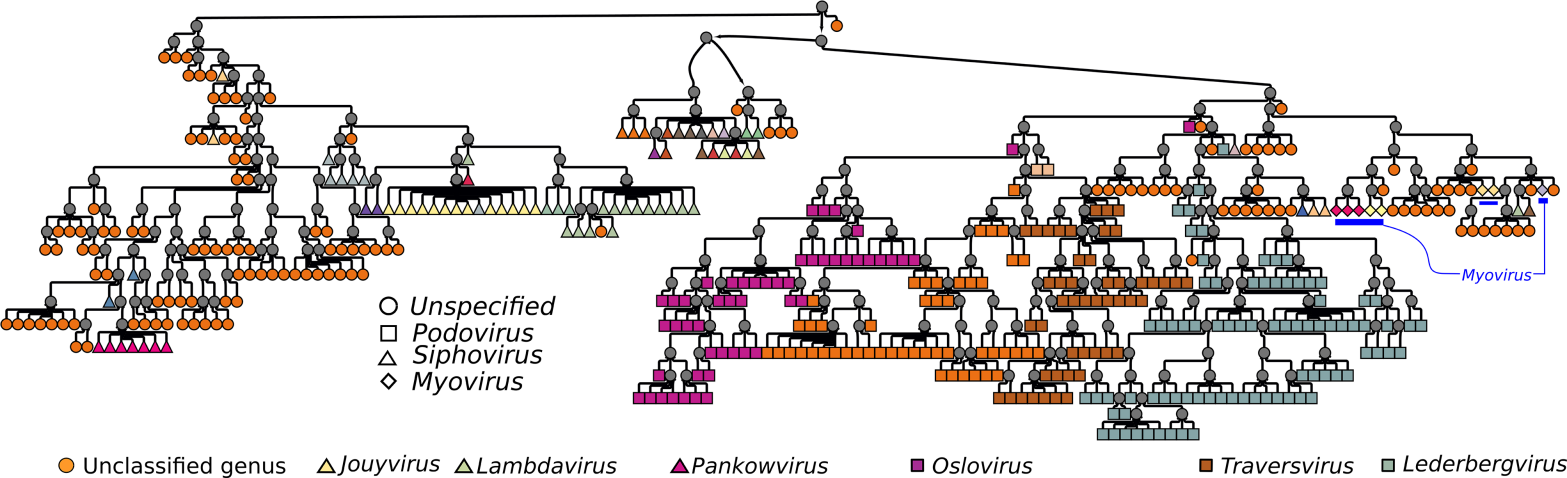
HieVi Lambdoid branch. Viruses are coloured by ICTV genera. Reported virion morphology: (○) Unspecified, (□) Podovirus, (▱) Siphovirus, (◊) Myovirus. Highlighted genera: *Jouyvirus* (type phage Gifsy), *Lambdavirus* (type phage Lambda), *Pankowvirus*, *Oslovirus* (type phage TL2011), *Traversvirus* (type phage 933W) and *Lederbergvirus* (type phage P22). A and B: branches regrouping mostly siphoviruses. C: branch regrouping mostly podoviruses. Myoviruses are underlined by blue lines. Relevant annotations are summarized in the Supplementary Excel file HieVi_annotations.xlsx.

We identified in the HieVi hierarchical tree an interesting branch clustering 470 phages, 348 of which code for a predicted integrase necessary (but not sufficient) for a temperate lifestyle (Supplementary Table S1). Kupcozk *etal*. recently investigated co-transferred genes in lambdoid phages and defined 12 groups. Within each of these 12 groups, gene content is highly similar while it is highly divergent across the 12 groups [14]. Among these, one can find well studied temperate phages such as *Lambdavirus* Lambda, *Byrnievirus* HK97 and *Lederbergvirus* P22. The INPHARED dataset we used in our study comprises 30 lambdoid genomes out of the 31 used by Kupcozk *et al.* (not counting their *E. coli* prophage dataset that is not included in the INPHARED dataset). Among these 30 lambdoid phages, 29 are included in this HieVi branch. We thus provisionally named this branch “Lambdoid branch” (Supplementary Table S1). This branch also includes 186 temperate phages out of the 190 included in our dataset annotated as Shiga Toxin 1- and Shiga Toxin 2- converting phages. The HieVi lambdoid branch is presented in Fig. 7 where phages are coloured by ICTV genera.

All phages in this branch are unclassified at the family level and this branch includes only two defined ICTV families, the *Sepvirinae* (including *Traversvirus* type phage 933W) and *Hendrixvirinae* (including *Byrnievirus* type phage HK97). As we observed previously in other HieVi branches, phages are well clustered according to the ICTV genus (Fig. 7) and the two ICTV subfamilies (data not shown). Interestingly, this branch contains different virion morphologies. For example, *Escherichia* phages HK97 and Lambda exhibit siphovirus morphology (long, flexible, non-contractile tail), *Salmonella* phage P22 and *Escherichia phage* HK620 are podoviruses (short, non-contractile tail) and *Edwardsiella* phage GF-2 is a myovirus (long, contractile tail). In Fig. 7, we can observe that phages in the Lambdoid branch cluster rather well according to their reported morphology: siphoviruses (e.g. *Lambdavirus*, *Jouyvirus* and *Hendrixvirinae* phages) are mostly clustered in branch C, while podoviruses (e.g. *Sepvirinae* and *Lederbergvirus* phages) are mostly clustered in branches A and B. The eight reported myoviruses are nested within branch C.

Lambdoid phages have been well studied for decades and from these emerged the concept of mosaic genomes where genes are clustered in functional units that can be recombined between phages. As mentioned in the Introduction, this makes it difficult to trace the evolutionary trajectory of entire genomes. It is thus surprising that HieVi clustered all these lambdoid phages within a branch despite the gene content divergence across the 12 groups underlined by Kupcozk et *al*. and with phages exhibiting various virion morphologies and a wide range of genome sizes (40–70 kb). We posit that HieVi can capture evolutionary relationships that extend beyond the genus and subfamily taxonomic ranks and probably offers the opportunity to identify recombinant groups.

### HieVi use-case for query genomes

**Figure 8.**
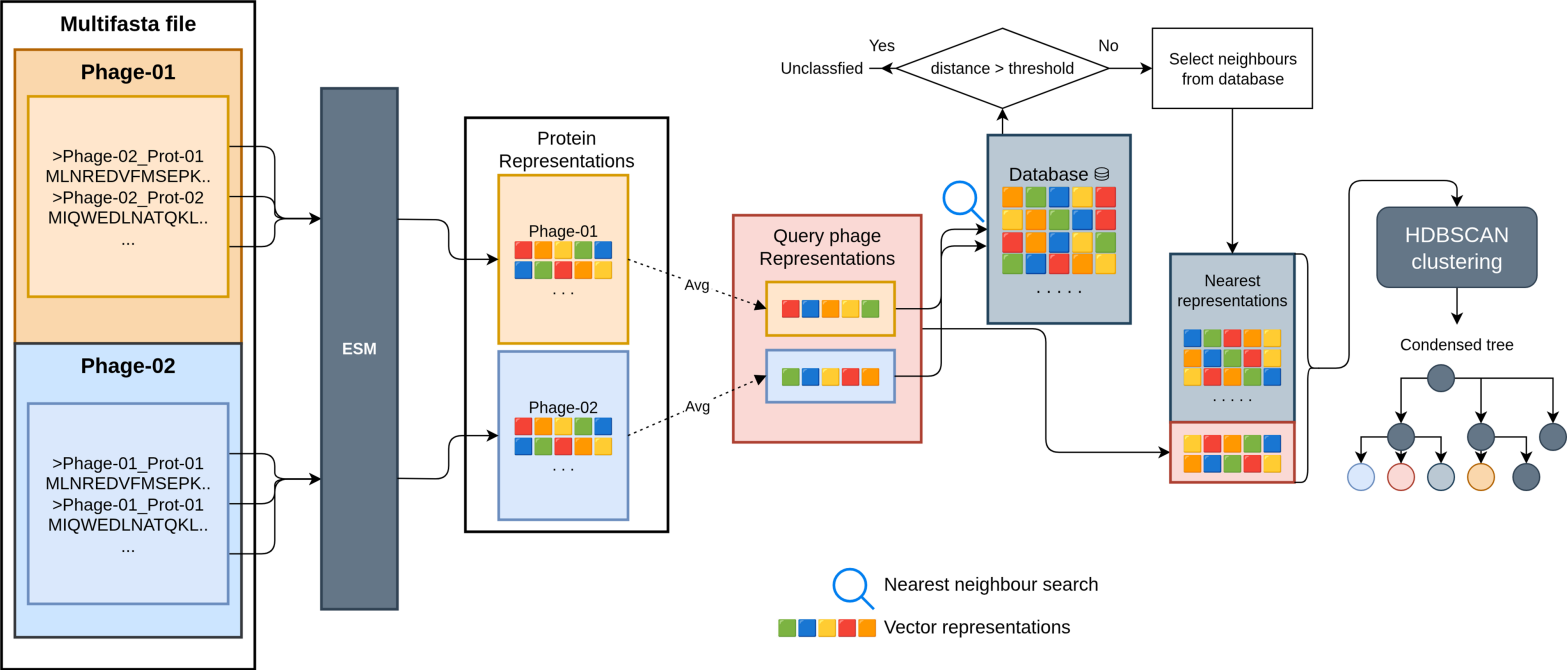
HieVi workflow for new proteomes. New viral proteins are first converted into vector representations with ESM-2 3B then normalized to unit length. MPRs for each query virus is then generated by averaging the protein representations within each proteome, respectively. For each phage representation we search and retrieve the nearest neighbours from the precomputed HieVi phage representation database. If the distance to nearest neighbour is farther than a threshold the phage is deemed unclassified otherwise the phages in the neighbourhood of the HieVi tree are selected. Then we re-run hierarchical clustering with HDBSCAN with the subset of phages and finally generate the condensed tree.

The HieVi database comprises the vector representations of all viruses included in the INPHARED dataset (September 2024 release). This precomputed vector database can be leveraged to identify new phages by comparing their vector representation in the HieVi database. The schematic of the workflow is shown in Fig. 8.

Firstly, we generate the vector representations of each protein in a query phage proteome, which are then normalized to unit-length and averaged to obtain a MPR for the respective query phage. Secondly, for each computed phage representation, the HieVi vector database is searched for nearest neighbours. If the first nearest neighbour has a distance above a threshold, then we assume that the query phage is too distant from all phages in the HieVi precomputed database and will be qualified as ‘unclassified’. Otherwise, the phage representations of the identified nearest neighbours are extracted, concatenated with the query phage representations and then clustered using HDBSCAN. This allows to extract a subset of phages from the entire HieVi hierarchical tree displayed in Fig. 3B and generate a smaller relational hierarchical tree including the query phages. This condensed tree structure facilitates an approximate positioning of the query phages relative to their nearest phages in the dataset without relying on annotations.

This approach avoids the need to reprocess all existing genomes in the HieVi database, thus distinguishing it from methods relying on Multiple Sequence Alignments such as vConTACT v2.0. This feature highlights the method's scalability potential, provided that the vector database can be updated with new sequences efficiently.

It is worth reiterating that the efficiency of this workflow is contingent upon the quality and completeness of the input genomes as well as the existence of close relatives in the precomputed dataset. We envisage augmenting the database diversity with complete viral sequences issues from various metagenomic datasets and prophages systematically mined into published prokaryotic genomes (e.g. GenBank, RefSeq or ENA) but also in bacterial genomes and metagenomes.

### Limitations

The HieVi framework, while innovative, has its limitations that are important to acknowledge for a comprehensive understanding of its scope and application. The framework considers a phage as a bag of proteins. Even though such a construction is simple and computationally efficient (averaged representations), it leads to limitations such as biological interpretation. We list some of the limitations below.

#### Dataset composition and biases

Phage classification and taxonomy, especially at intermediate taxonomic ranks, are constantly evolving alongside biologically interpretable tools. As mentioned earlier, complete and well annotated datasets such as INPHRAED are already biased in their content by the phages that were isolated experimentally and this limits the ability to quantify the accuracy of generalized and supervised classification algorithms. Training supervised classification algorithms on highly unbalanced datasets can limit their generalizability. Consequently, in this study, we resorted to using unsupervised methods to hierarchical organization emerging from MPRs constructed from pre-trained pLMs.

#### Metagenomic data and partial genomes

For reliable placement of phage representations, complete and high-quality genome sequences are required. Partial or noisy contigs resulting from poor DNA sequencing quality can significantly lead to modified MPRs due to poor or erroneous syntactic annotation. For instance, certain “false positives” in *Autographiviridae* branches, such as some *Kayfunavirus* and *Tunavirinae* phages, were identified as stemming from poor DNA sequencing quality.

#### Distance contrasts

As inferred from the mathematical description we provide in Supplementary Information II, the MPR norms encode functional diversity and dot-products encode shared protein family information. However, the inter-protein family terms can be significant and not tend to zeros when the embeddings are not fine-tuned, leading to low contrast in the distances. Moreover, in high dimensions, as is the case with the pre-trained ESM-2 3B we used, the distances tend to homogenize and learned embeddings can have angularly tight distributions. Therefore, fine-tuning with smaller models with less than 3 billion parameters may help increase efficiency in both computation and contrast between vectors.

#### EPS and distance thresholds

The ongoing efforts to identify strict and well-defined rules for demarcation of subfamily and family and higher levels in taxonomy introduce a challenge to allow for identification of a clear eps/distance threshold for each taxonomic rank. This is supported by the histogram Euclidean distance within and between taxa plotted in Supplementary Information I Supplementary Fig. S6. To construct the histogram, we first exclude the realm *Monodnaviria* which has different criteria for taxonomic ranks. Then we randomly pick 10 000 pairs within genus and between genera phages and construct the histogram of Euclidean distance. For within-between distance histogram at genus level, there is a clear distinction with a best threshold at 0.0006, leading to a True Positive Rate (TPR) of 0.98 and a False Positive Rate (FPR) of 0.11. At the family level, we see a bimodality in the within family distance histogram with best threshold at 0.059 and a TPR of 0.87 and FPR of 0.08. Thus, extracting flat clusters that homogeneously corresponds to ICTV taxonomy is not feasible yet. We defer this to future work to reliably identify the criteria and eps/distance thresholds and the biological criterion arising from such a new hierarchy.

### Scalable organization but not directly interpretable

It is also important to note that HieVi is distinct from approaches such as vConTACT v2.0. While vConTACT offers interpretable community-based clustering using shared PCs, it is less scalable to very large datasets. In contrast, HieVi provides a scalable framework that facilitates exploration of proteomic similarities across genomes but remains relatively less interpretable, for example, in identifying core genes. We also acknowledge that viral evolutionary histories can be non-hierarchical and graph-like in nature, thus, HieVi should be viewed as a complementary tool rather than a definitive tool for deriving taxonomy directly. For instance, it can be used to filter or organize datasets prior to downstream analyses with tools such as vConTACT v2.0 and VIRIDIC. Together, these approaches can support a more nuanced and scalable phage taxonomy pipeline.

## Discussion

In this work, we explore the application of pLMs for the comparison and classification of phages, presenting a novel approach which extends the repertoire of proteome-based analysis of phages. We introduced *Hierarchical Viruses* or HieVi, a method that has the potential to address the increasing need for managing and organizing large datasets of phage genomes beyond multiple sequence alignment methods.

We showed that protein embeddings from a self-supervised, pre-trained pLM when averaged can encode evolutionary information for phage taxonomy even without any supervision or fine-tuning. The advantage of such vector representations is evident in the context of scalable vector databases and fast nearest neighbour searches, which are necessary for ever growing datasets.

We show that the vectorial representation of phages derived from embeddings from pLMs encodes average function of the genes and distance between the MPRs in this space contains information about both functional diversity and shared functions. By using hierarchical clustering and extracting the condensed tree we show that the hierarchical relations between phages are maintained by the phage representation vectors that align well with current ICTV annotation (Fig. 3). Through selected *Caudoviricetes* phage families—the *Herelleviridae*, *Straboviridae*, and *Autographiviridae*—we first qualitatively observed that phages are grouped in the HieVi hierarchical tree topology along their ICTV genera and subfamilies (Figs 3–5 and 8). Clustering efficiency metrics quantitatively supported these observations (Fig. 4 & Supplementary Fig. S2). Phages belonging to the same ICTV family are also grouped in the tree topology (Fig. 3), with the notable exception of *Autographiviridae*. Existing phylogeny within families has been defined using various approaches, the most recent one relying on whole genome/proteome analyses. Since the HieVi hierarchy is generally in good accordance with these established phylogenies, we posit that this hierarchy encodes phylogenetic relationships, at least up to the family level and can assist biologists to discover and define new phage families.

The *Autographiviridae* family, essentially defined by the presence of a gene encoding a large single subunit RNA polymerase, is an illustrative example of how the HieVi hierarchical tree topology can reveal unsuspected complexity within a taxon. Previous studies have evidenced the diversity of this family, with 130 genera and eight subfamilies. At variance with other phage families, the *Autographiviridae* does not appear as a single branch in the HieVi tree topology but is split into nine distinct branches. Within each branch, genus and subfamily clustering is respected as evidenced in other phage families, suggesting that each branch represents a phylogenetic unit on its own (Fig. 5). Combining tree topology, functional annotation (e.g. the presence of an integrase) and other genomic features (*e.g*. genome size), the nine HieVi branches encompassing the *Autographiviridae* can serve as a blueprint for phage taxonomists to define nine new monophyletic taxa regrouped at a new, higher taxonomic rank, the *Autographivirales* order. Interestingly, during the course of this manuscript preparation, ICTV recently ratified (February 2025) a proposal creating the *Autographivirales* order, comprising four new families (ICTV proposal 2024.045B), which is in line with our findings. These new considerations for *Autographivirales* and new families were obtained using multiple approaches described before. In light of this recent development, we provide Supplementary Table S2 in Supplementary Information I comparing HieVi branches for *Autographiviridae* phages (Table 4) and the new ICTV classification. This recent ICTV update nicely confirms our own findings regarding *Autographiviridae* phages but also gives weight to using HieVi framework for defining robust new classification at least up to the family level. However, accurately modelling at the rank of order like the *Autographivirales* and the new *Pantevenvirales* (also ratified in February 2025) remains a topic for future work focused on improving the interpretability of MPRs.

Latest phage phylogeny relies on the convergence of several whole genome/proteome analyses as exemplified by the *Herelleviridae* family [15]. Thus, we demonstrate that HieVi enables the emergence of a multi-scale taxonomic organization of phages. The organization of the lambdoid branch we defined suggests that the HieVi hierarchical tree captures more than just shared genes (Fig. 7). With this example, we showed that HieVi transcends conventional gene-sharing metrics and highlights evolutionary insights into the gene flux resulting in phage genome mosaicism.

We thus hypothesize that pLM-based phage comparison and classification enabled by HieVi enhance our ability to discover and interpret evolutionary patterns, and possibly phylogeny-driven view of phage diversity. Large proteomic datasets, with ever-increasing additions due to global efforts in viral ecology, require better data organization. Here, we used a large pLM (ESM-2 with 3 billion parameters) and showed the emergence of phage hierarchy from protein embeddings-based phage representation. While larger models are known to capture the evolutionary and semantic structure well, they require substantial computational resources (high GPU memory ∼24 GB to 32 GB) and have high embedding dimensions (*n =* 2560-dimension vectors). Smaller pLMs inherently have relatively lower embedding dimensions and offer advantages in computational and storage costs but they come with trade-offs. Smaller models may not capture the full complexity of semantic or evolutionary signals as effectively as larger ones, particularly for diverse or rare protein families.

We also provide a mathematical formulation of the construction of MPRs showing how the distances may be related to the functional diversity of phages and the shared functions. Accordingly, we can infer that a preferred alternative would be to fine-tune smaller models (with lower dimensions) on viral proteins, which could help separate and orthogonalize protein families, leading to larger distances between MPRs, in contrast with pre-trained models that are compact in the embedding space (Supplementary Fig. S7). Such benchmarking with smaller models or fine-tuning represents an intriguing and valuable future direction for developing more computationally accessible versions of HieVi.

Conceptually, this study builds on the advancements seen in pLMs and the ESM Metagenomic Atlas published only 2 years ago [7]. pLMs have shown an impressive ability to learn the ‘language’ of proteins in a self-supervised manner, providing insights into their functions and evolutionary relationships [35]. HieVi extends this capability to whole proteomes, which to our knowledge has never been explored so far, demonstrating that averaged vector representations of proteomes can capture the evolutionary relationships between phages for comparison and classification. Ultimately, HieVi equips researchers in the field of phage studies with a well-suited, dedicated and complimentary tool for organizing, classifying, and exploring the complex and ever-growing landscape of viral ecology, paving the way for future discoveries.

## Supplementary Material

lqaf134_Supplemental_Files

## Data Availability

Phage representation vectors in Zarr format generated from the INPHARED dataset are available at https://drive.google.com/file/d/1vC07hjmbNBXsPIW5R1FUIxLwrDvb3BP9/view?usp=sharing. HieVi UMAP Phage Atlas September 2024 release (Fig. 1) can be interactively viewed at https://pswapnesh.github.io/HieVi/HieVi_UMAP.html. HieVi workflow and code are available at Zenodo (https://doi.org/10.5281/zenodo.17018992) and Github (https://github.com/pswapnesh/HieViSearch/). Single genome comparison is available as demo at https://huggingface.co/spaces/pswap/hievi. Due to the large file size, the vConTACT v2.0 similarity network (Supplementary Fig. S2) is available on request. vConTACT v2.0 clustering results are included in HieVi annotation Excel file (HieVi_annotations.xlsx).
