# Supplementary material for: Phage evolutionary relationships emerge from protein language model-based proteome representation
Source: NAR Genom Bioinform. 2025 Oct 22;7(4):lqaf134. doi: 10.1093/nargab/lqaf134 (PMC12541379; doi:10.1093/nargab/lqaf134)
Supplement: lqaf134_Supplemental_Files [file lqaf134_supplemental_files.zip › Supplementary_information_II_R2.pdf]

Supplementary Information II for Phage evolutionary relationships emerge from protein Language Model-based proteome representation

Swapnesh Panigrahi, Mireille Ansaldi and Nicolas Ginet

Phage cycle and bacterial metabolism team - Laboratoire de Chimie Bactérienne - UMR7283 CNRS/Aix-Marseille Université, Marseille, 13009, France

Embedding from language models captures semantic meaning by positioning similar concepts close together in high-dimensional angular space. In protein language models like ESM-2, it has been shown that the learning encodes alignment with protein families [1] and the embeddings form structured clusters that correspond to protein families, reflecting functional and evolutionary characteristics.

Here we formalize the construction of the mean phage representation (MPR) in order to provide insights into the information encoded in the MPRs. Let the embeddings from the protein language model (e.g., ESM-2) be normalized to lie on the unit hypersphere $\mathbb{S}^{d-1} \subset \mathbb{R}^d$. These learned embeddings from the pre-trained protein language model (pLM) are tightly clustered in angular space. Under the hypothesis that they form a structured spaces with clusters corresponding protein families (as indicated in [1]), let us assume the normalized protein embeddings, $\mathbf{u}_1, \ldots, \mathbf{u}_m \in \mathbb{R}^d$, are drawn from a mixture of K clusters or distributions, each corresponding to a protein family. For family k, let the distribution have mean direction $\boldsymbol{\mu}_k$ and concentration α_k. Let the expectation of a vector drawn from the family k be $\mathbb{E}[\mathbf{u}_i] = \alpha_k \boldsymbol{\mu}_k$, then $\mathbb{E}[\mathbf{u}_i^T \mathbf{u}_j] = \alpha_k^2$, for independent i, j in same family k, where α_k is related to the semantic tightness of the cluster k. Thus α_k^2 is the mean cosine of vectors within the cluster k.

Mean phage representation

We treat a phage as a bag of proteins given by a set of normalized protein embeddings $\Phi = \{\mathbf{u}_1, \ldots, \mathbf{u}_m\}$ and define its average embedding as:

$$\boldsymbol{\phi} = \frac{1}{m} \sum_{i=1}^{m} \mathbf{u}_i.$$

We normalize the protein embeddings to only retain the functional direction, so that conserved proteins (with larger norms) do not dominate the mean. In this case, all the genes and thier functions in a phage contribute equally to the mean phage representation. Then we can write the expected squared-norm of the MPR as:

$$\mathbb{E}\left[\|\boldsymbol{\phi}\|^2\right] = \mathbb{E}\left[\left(\frac{1}{m}\sum_{i=1}^{m} \mathbf{u}_i\right)^T \left(\frac{1}{m}\sum_{j=1}^{m} \mathbf{u}_j\right)\right] = \frac{1}{m^2} \sum_{i=1}^{m} \sum_{j=1}^{m} \mathbb{E}[\mathbf{u}_i^\top \mathbf{u}_j].$$

The expected dot product can be written as, $\mathbb{E}[\mathbf{u}_i^\top \mathbf{u}_j] = \alpha_{k(i)} \alpha_{k(j)} \boldsymbol{\mu}_{k(i)}^\top \boldsymbol{\mu}_{k(j)}$, such that the normalized protein embedding $\mathbf{u}_i$ belongs to a family $k(i)$. Now, we define two sets of indices, namely, intra-family and inter-family sets as :

$$\mathcal{I} = \{(i,j) : k(i) = k(j)\}, \quad \mathcal{E} = \{(i,j) : k(i) \neq k(j)\}.$$

Then the expected square norm can be written as:

$$\mathbb{E}\left[\|\boldsymbol{\phi}\|^2\right] = \frac{1}{m} + \frac{1}{m^2} \sum_{(i,j)\in\mathcal{I}} \alpha_{k(i)}^2 + \frac{1}{m^2} \sum_{(i,j)\in\mathcal{E}} \alpha_{k(i)} \alpha_{k(j)} \boldsymbol{\mu}_{k(i)}^\top \boldsymbol{\mu}_{k(j)}, \quad (1)$$

where the first term is the "diagonal" term when $i = j$ and $\mathbf{u}_i^T \mathbf{u}_i = 1$. In the case where all the genes are from different families, we have

$$\mathbb{E}\left[\|\boldsymbol{\phi}\|^2\right] = \frac{1}{m} + \frac{1}{m^2} \sum_{(i,j)\in\mathcal{E}} \alpha_{k(i)} \alpha_{k(j)} \boldsymbol{\mu}_{k(i)}^\top \boldsymbol{\mu}_{k(j)}. \quad (2)$$

Representing the mean functional diversity by δ, we write,

$$\mathbb{E}\left[\|\boldsymbol{\phi}\|^2\right] \sim \frac{1}{m} + m(m-1)\frac{1}{m^2}\delta \tag{3}$$

$$= \frac{1}{m} + \left(1 - \frac{1}{m}\right)\delta. \tag{4}$$

In the case of random vectors where the embeddings tend to be orthogonal $\delta \to 0$, leading to decrease in norm with number of averaged vectors. However, learned embeddings from pre-trained models are compact in angular space leading to finite contribution from this term, even when the genes are from different families. In our case, where we use pre-trained ESM-2 model, the MPRs remain compact as shown in the Figure S7.A with norms close to 1 (Figure S7.C). The norm is also plotted against the genome length (Figure S7.B) to show that the norm doesn't significantly decrease with increase in the genome length. Fine-tuning a model so that the embeddings have contrast, with dot products between nearly orthogonal families tend to zero. Thus, as m increases the expected norm reflects the gene diversity, while for smaller genomes it is close to 1.

Alternatively, grouping by family, we write:

$$\mathbb{E}[\boldsymbol{\phi}] = \sum_{k=1}^{K} \frac{n_k}{m}\alpha_k \boldsymbol{\mu}_k, \quad \mathbb{E}[\|\boldsymbol{\phi}\|^2] = \frac{1}{m^2}\sum_{k=1}^{K}\sum_{l=1}^{K} n_k n_l \alpha_k \alpha_l \boldsymbol{\mu}_k^\top \boldsymbol{\mu}_l. \tag{5}$$

Comparing phages

Considering two phages $\Phi_u = \{\mathbf{u}_1, \dots \mathbf{u}_{m_u}\}$ and $\Phi_v = \{\mathbf{v}_1, \dots \mathbf{v}_{m_v}\}$, represented by the MPRs $\boldsymbol{\phi}_u$ and $\boldsymbol{\phi}_v$, we may write the expectation of the dot product as

$$\mathbb{E}[\boldsymbol{\phi}_u^T \boldsymbol{\phi}_v] = \mathbb{E}\left[\left(\frac{1}{m_u}\sum_{i=1}^{m_u}\mathbf{u}_i\right)^T \left(\frac{1}{m_v}\sum_{i=1}^{m_v}\mathbf{v}_j\right)\right] \tag{6}$$

$$\mathbb{E}[\boldsymbol{\phi}_u^T \boldsymbol{\phi}_v] = \frac{1}{m_u m_v}\sum_{i=1}^{m_u}\sum_{j=1}^{m_v}\mathbb{E}[\mathbf{u}_i^T \mathbf{v}_j] \tag{7}$$

Assuming independence between $\mathbf{u}_i$ and $\mathbf{v}_j$, we may write

$$\mathbb{E}[\mathbf{u}_i^T \mathbf{v}_i] = \mathbb{E}[\mathbf{u}_i]^T \mathbb{E}[\mathbf{v}_j] = \alpha_{k(i)}\alpha_{k(j)}\boldsymbol{\mu}_{k(i)}^T \boldsymbol{\mu}_{k(j)}.$$

$$\mathbb{E}[\boldsymbol{\phi}_u^T \boldsymbol{\phi}_v] = \frac{1}{m_u m_v}\left(\underbrace{\sum_{k(i)=k(j)} \alpha_{k(i)}^2}_{\text{shared protein families}} + \sum_{k(i)\neq k(j)} \alpha_{k(i)}\alpha_{k(j)}\boldsymbol{\mu}_{k(i)}^\top \boldsymbol{\mu}_{k(j)}\right). \tag{8}$$

Grouping by families, let n_k^u be the number of vectors in Φ_u from a family cluster k and n_l^v be the number of vectors in Φ_v from a family cluster l. Then:

$$\mathbb{E}[\boldsymbol{\phi}_u^T \boldsymbol{\phi}_v] = \frac{1}{m_u m_v}\sum_{k=1}^{K}\sum_{l=1}^{K} n_k^u n_l^v \alpha_k \alpha_l \mu_k^T \mu_l$$

Splitting the general term into intra-family ($k = l$) and inter-family ($k \neq l$) terms, we have:

$$\mathbb{E}[\boldsymbol{\phi}_u^T \boldsymbol{\phi}_v] = \frac{1}{m_u m_v} \left(\underbrace{\sum_{k=1}^{K} n_k^u n_l^v \alpha_k^2}_{k=l, intra-family} + \underbrace{\sum_{k=1}^{K} \sum_{l=1}^{K} n_k^u n_l^v \alpha_k \alpha_l \mu_k^T \mu_l}_{k \neq l, inter-family} \right) \tag{9}$$

Euclidean distance

$$\mathbb{E}[\|\boldsymbol{\phi}_u - \boldsymbol{\phi}_v\|^2] = \underbrace{\mathbb{E}[\|\boldsymbol{\phi}_u\|^2] + \mathbb{E}[\|\boldsymbol{\phi}_v\|^2]}_{\text{gene diversity}} - 2 \underbrace{\mathbb{E}[\boldsymbol{\phi}_u^T \boldsymbol{\phi}_v]}_{\text{shared genes}}$$

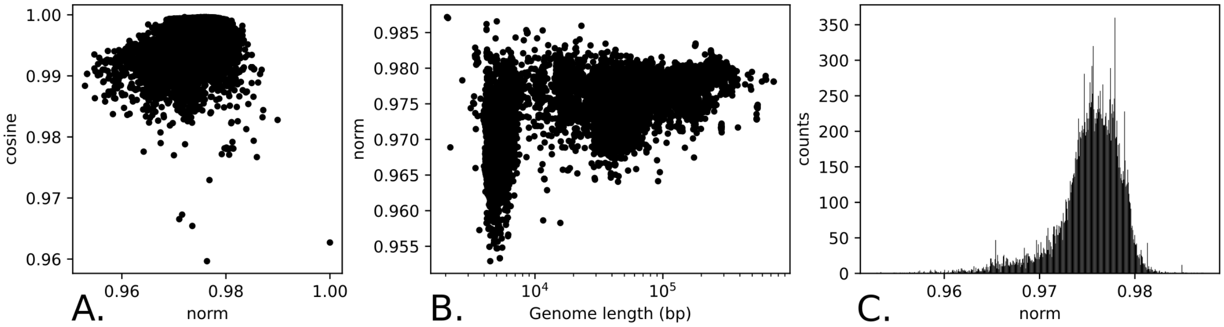

Figure S7: **Norm and angular compactness of the MPRs**. A. The MPRs are compact in the embedding space. The cosine between the each vector and their mean is plotted against the norm of the corresponding vector. B. The norm is plotted against the genome length. C. The histogram of the norms of the MPRs.

Reference

[1] Alexander Rives, Joshua Meier, Tom Sercu, Siddharth Goyal, Zeming Lin, Jason Liu, Demi Guo, Myle Ott, C. Lawrence Zitnick, Jerry Ma, and Rob Fergus. *Biological structure and function emerge from scaling unsupervised learning to 250 million protein sequences.* Proceedings of the National Academy of Sciences, 118(15):e2016239118, 2021. DOI: 10.1073/pnas.2016239118
