# Supplementary material for: Phage evolutionary relationships emerge from protein language model-based proteome representation
Source: NAR Genom Bioinform. 2025 Oct 22;7(4):lqaf134. doi: 10.1093/nargab/lqaf134 (PMC12541379; doi:10.1093/nargab/lqaf134)
Supplement: lqaf134_Supplemental_Files [file lqaf134_supplemental_files.zip › Supplementary_information_I_R2.docx]

**SUPPLEMENTARY INFORMATION I**

**Phage evolutionary relationships emerge from protein Language Model-based proteome representation**

Panigrahi Swapnesh, Ansaldi Mireille and Ginet Nicolas

**FIGURES**

**
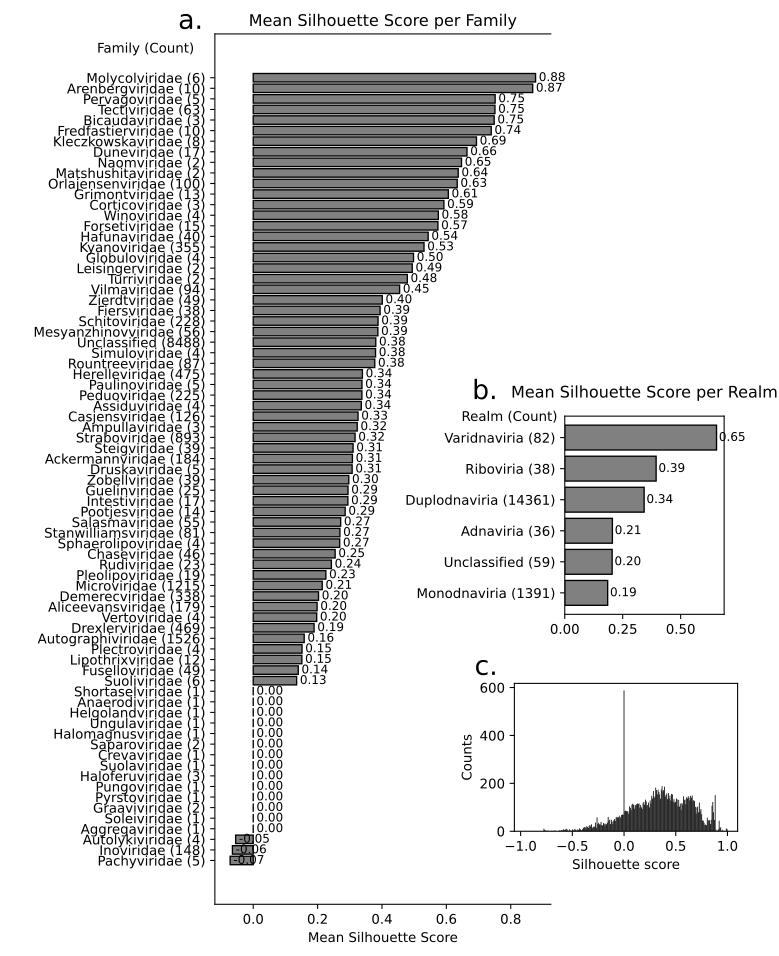
**

**Figure S1: Silhouette scores.** The plot shows the mean Silhouette scores of MPRs to ICTV genus. a) Shows the mean Silhouette scores of the phages within each family with the number of phages in each family shown next to the family name. Mean Silhouette score is 0 for under-represented families. b) Shows the mean Silhouette scores of samples belonging to different realms, with *Monodnaviria* showing the lowest mean Silhouette. c) Shows the histogram of per sample Silhouette score. Some genera are under-represented leading to low Silhouette score. Genera with mean Silhouette score less than 0.2 have less than three phages in them.


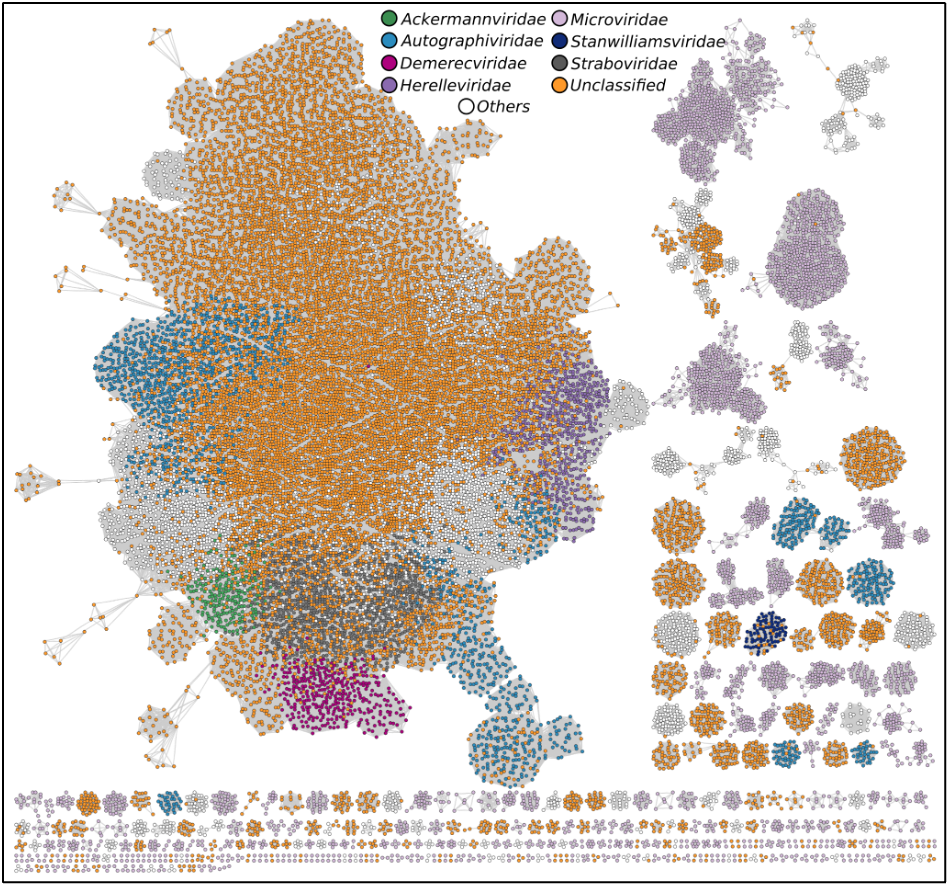


**Figure S2: vConTACT v2.0 Phage Network.** *Straboviridae*, *Herelleviridae* and *Autographiviridae* as well as several other ICTV families are colored (see legend). Phages belonging to other ICTV families are colored in white. Unclassified families are colored in orange. Relevant annotations are summarized in the Supplementary Excel file HieVi_annotations.xlsx.


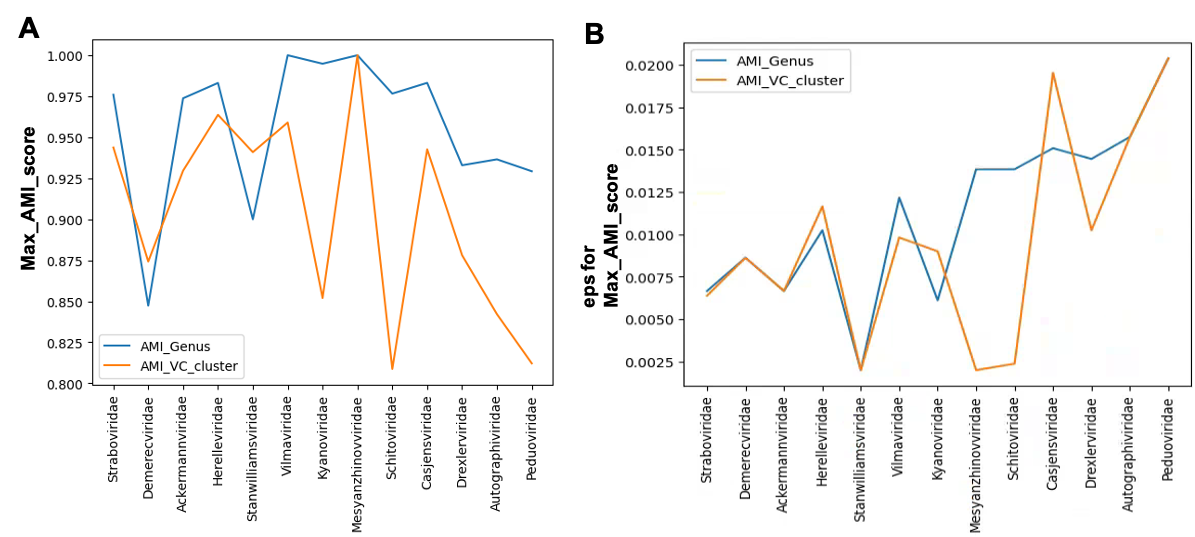


**Figure S3: HieVi clustering efficiency across 13 ICTV families.** A) AMI maximum score for ICTV genus (blue line) and vConTACT v2.0 VCs (orange line). B) eps distance threshold for maximum AMI score for ICTV genus (blue line) and vConTACT v2.0 VCs (orange line).


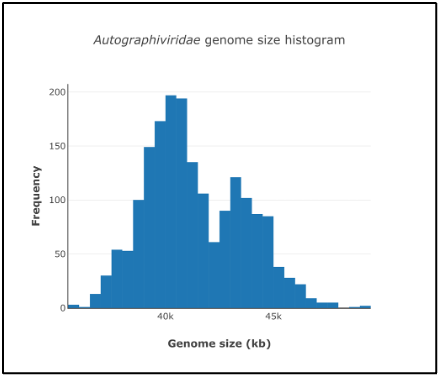


**Figure S4: *Autographiviridae* genome size distribution.** Genome size histogram for *n* = 1,874 phages included in *Autographiviridae* branches (Table 4). Bin size = 0.5 kb. Genome sizes are summarized in the Supplementary Excel file HieVi_annotations.xlsx.


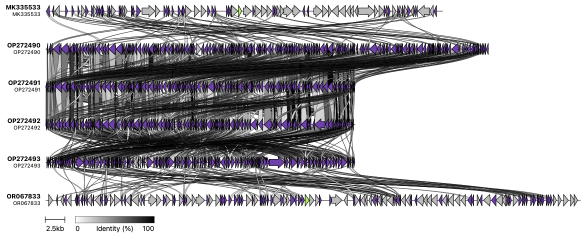


**Figure S5: *Autographiviridae* branch 09 sequence alignment.** MK335533: *Shigella* phage vB_SsoS_008 (*Drexlerviridae*, *Tunavirinae*, *Tunavirus*). OP272490 to OP272493: *Enterobacter* phages (*Autographiviridae*, *Studiervirinae*, *Kayfunavirus*). OR067833: *Serratia* phage KKP 3709 (*Unclassified*, *Unclassified*, *Myosmarvirus*). Sequence alignment generated by Clinker (amino acid sequence identity threshold = 0.225) (1).


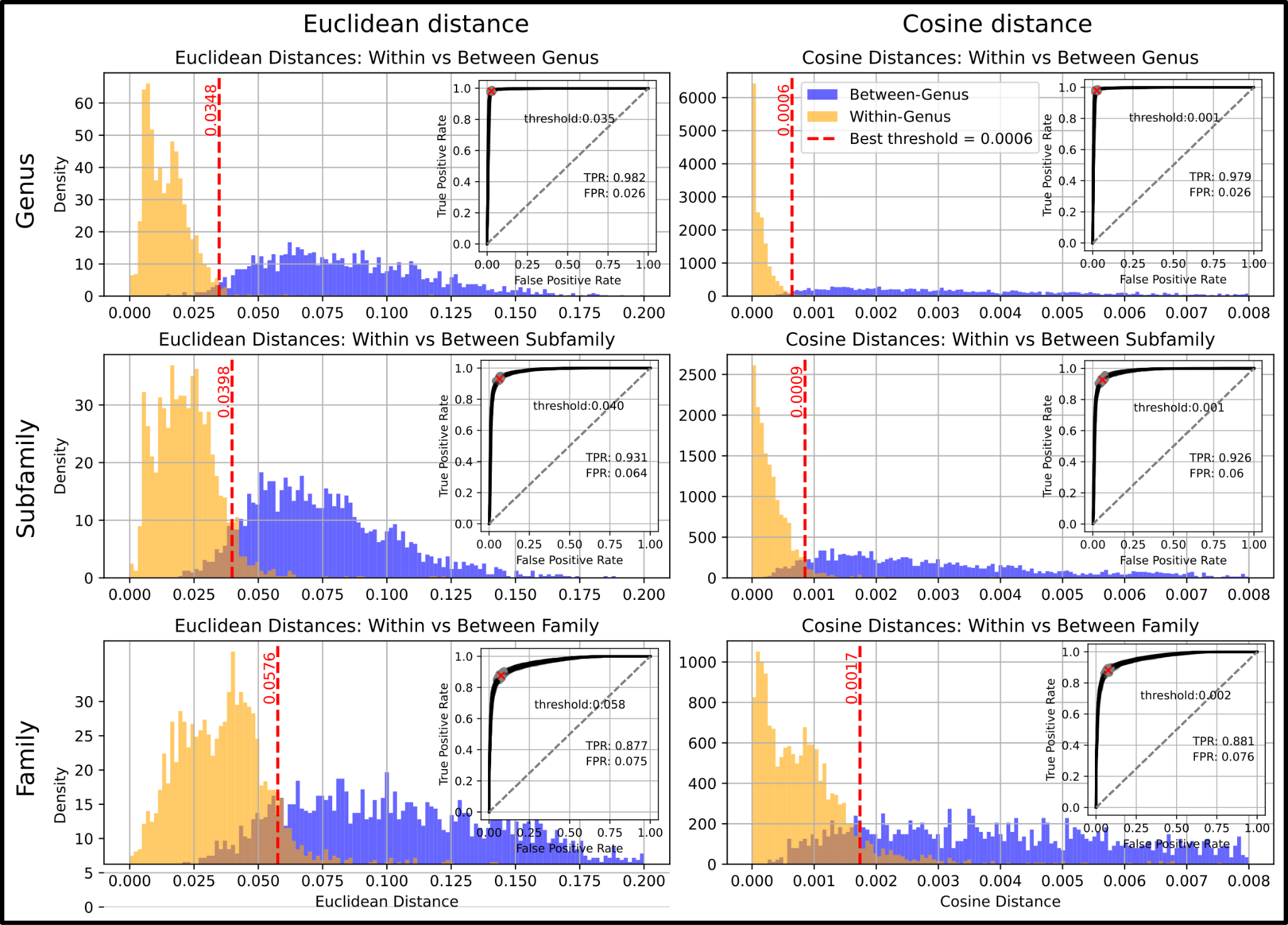


**Figure S6:** **Histogram of within and between-taxa distances**. The Figure shows the within, and between taxa (genus, subfamily and family) distance histograms with corresponding Receiver Operating Characteristic (ROC) curves and in the inset optimal distance thresholds detected at maximum of True Positive Rate (TPR) - False Positive Rate (FPR). First, we exclude the realm *Monodnaviria* as the criteria for taxonomy differs from the others. Then, we pick *n* = 2,048 pairs of phages at random from the same genus and from different genera and plot the histogram of Euclidean distances (left) and cosine distances (right) between the pairs of phages. The inset ROC curve is obtained by scanning the thresholds of distance for classification of the pairs as “within” or “between”. The mean threshold obtained out of 32 runs is indicated on the histogram.

**TABLES**

| **Branch** | **Unclassified family** | **Subfamily** | **Main host order** |
| --- | --- | --- | --- |
| 01 (470) | 470 | *Hendrixvirinae* (18), *Sepvirinae* (150), Unclassified (302) | *Enterobacterales* (454) |

**Table S1: Lambdoid branch annotations.** This branch comprises 32 ICTV genera (204 phages are unclassified at the genus level).

| **Branch** | **Family** | **Subfamily** | **Genus** |
| --- | --- | --- | --- |
| 01 (266) | *Autotranscriptaviridae* (266) | *Studiervirinae* (263), Unclassified (3) | Too many to list |
| 02 (243) | *Autonotataviridae* (57), *Autoscriptoviridae* (125), *Autosignataviridae* (44), Unclassified (17) | *Gujervirinae* (25), *Melnykvirinae* (15), *Okabevirinae* (2), *Corkvirinae* (2), *Krylovirinae* (17), *Slopekvirinae* (84) , Unclassified (37) | Too many to list |
| 03 (51) | *Autoscriptoviridae* (51) | *Beijerinckvirinae* (46), *Slopekvirinae* (4), Unclassified (1) | *Aristophanesvirus* (1), *Friunavirus* (45), *Kakivirus* (1), *Novosibovirus* (3), *Linggongvirus* (1) |
| 04 (5) | Unclassified (5) | *Sechaudvirinae* (5), Unclassified (1) | *Angmobvirus* (1), *Dishuivirus* (1), *Nerivirus* (1), *Spiovirus* (1), *Tiranvirus* (1) |
| 05 (3) | Unclassified (3) | Unclassified (3) | *Aequorvirus* (1), *Fussvirus* (1), *Thoosavirus* (1) |
| 06 (7) | *Autoscriptoviridae* (7) | *Stentvirinae* (7) | *Bonnellvirus* (7) |
| 07 (6) | Unclassified (6) | Unclassified (6) | *Boesrvirus* (1), *Chamilpavirus* (1), *Gyeongsanvirus* (1), *Paadamvirus* (1), *Pastovirus* (1), *Vistulavirus* (1) |
| 08 (9) | Unclassified (9) | Unclassified (9) | *Actaeavirus* (1), *Dynamenevirus* (3), *Oceanidvirus* (2), *Shangxiadianvirus* (2), *Triteiavirus* (1) |

**Table S2: Updated *Autographiviridae* branches taxonomy.** We filtered accession numbers from Table 4 (phages belonging to the eight first *Autographiviridae* branches) to retain only those listed in ICTV 2024.045B proposal with their lineage updated. *n* = 590 accession numbers.

**REFERENCES**

1. Gilchrist CLM and Chooi YH. clinker & clustermap.js: automatic generation of gene cluster comparison figures. *Bioinformatics* 2021; 37: 2473–2475. <https://doi.org/10.1093/bioinformatics/btab007>.
